# On the Microstructure and Properties of Nb-12Ti-18Si-6Ta-5Al-5Cr-2.5W-1Hf (at.%) Silicide-Based Alloys with Ge and Sn Additions

**DOI:** 10.3390/ma13173719

**Published:** 2020-08-22

**Authors:** Jiang Zhao, Claire Utton, Panos Tsakiropoulos

**Affiliations:** Department of Materials Science and Engineering, Sir Robert Hadfield Building, The University of Sheffield, Mappin Street, Sheffield S1 3JD, UK; zhaojiang6325@hotmail.com (J.Z.); c.utton@sheffield.ac.uk (C.U.)

**Keywords:** Nb-silicide-based alloys, high entropy alloys, complex concentrated alloys, microstructures, oxidation, intermetallics, silicides, alloy design

## Abstract

The microstructures and properties of the alloys JZ3 (Nb-12.4Ti-17.7Si-6Ta-2.7W-3.7Sn-4.8Ge-1Hf-4.7Al-5.2Cr) and JZ3+(Nb-12.4Ti-19.7Si-5.7Ta-2.3W-5.7Sn-4.9Ge-0.8Hf-4.6Al-5.2Cr) were studied. The densities of both alloys were lower than the densities of Ni-based superalloys and many of the refractory metal complex concentrated alloys (RCCAs) studied to date. Both alloys had Si macrosegregation and the same phases in their as cast and heat treated microstructures, namely βNb_5_Si_3_, αNb_5_Si_3_, A15-Nb_3_X (X = Al, Ge, Si, Sn), C14-Cr_2_Nb and solid solution. W-rich solid solutions were stable in both alloys. At 800 °C only the alloy JZ3 did not show pest oxidation, and at 1200 °C a thin and well adhering scale formed only on JZ3+. The alloy JZ3+ followed parabolic oxidation with rate constant one order of magnitude higher than the single crystal Ni-superalloy CMSX-4 for the first 14 h of oxidation. The oxidation of both alloys was superior to that of RCCAs. Both alloys were predicted to have better creep at the creep goal condition compared with the superalloy CMSX-4. Calculated Si macrosegregation, solid solution volume fractions, chemical compositions of solid solution and Nb_5_Si_3_, weight changes in isothermal oxidation at 800 and 1200 °C using the alloy design methodology NICE agreed well with the experimental results.

## 1. Introduction

Refractory metal intermetallic composites (RMICs, list of abbreviations is given at the end of the paper) are candidate metallic materials to replace Ni-based superalloys in high pressure turbines to enable the latter to operate at temperatures significantly higher than those that are currently possible using Ni-based superalloys, and thus make it feasible for future aero-engines to meet strict environmental and performance targets [1]. Other metallic materials currently considered for similar applications are RHEAs, i.e., refractory metal (RM) high entropy alloys (HEA) and RM complex concentrated alloys (RCCAs) [2]. Nb-silicide-based alloys are the RMICs that attract much attention for blade applications owing to their low densities and capacity to offer a balance of properties [3,4].

Nb-silicide-based alloys are developed to meet the specific property goals (see next section) that were chosen for the new metallic materials. Major challenges for alloy developers have been how to design alloys that (a) do not suffer from pest oxidation at intermediate temperatures, (b) have tolerable oxidation resistance at intermediate and high temperatures, (c) form scales that do not spall off and meet (d) the toughness goal and (e) the creep goal. Research in progress [4,5] has indicated that it is unlikely for an alloy to meet simultaneously the oxidation and mechanical property goals, thus one further challenge for alloy developers is to design/select alloys that (f) offer a balance of mechanical properties with acceptable oxidation resistance. 

The alloy design methodology Niobium Intermetallic Composite Elaboration (NICE) [4], which has been developed in our research group, addresses (a) to (f) and was used in the research described in this paper (NICE does not consider fracture toughness, but it can predict (calculate) the vol.% Nb_ss_, which is important for the toughness of Nb-silicide-based alloys). This research was motivated by four key findings, namely (i) the superior creep of Nb-silicide-based alloys with RM additions compared with Ni-based superalloys [5,6,7], (ii) the elimination of pest oxidation when Sn or Ge are in synergy with Al and Cr additions [4,7,8,9,10,11,12], (iii) the suppression of pest oxidation at intermediate temperature with the simultaneous elimination of scale spallation at a high temperature when Al, Cr, Ge and Sn were all simultaneously present in a Nb-silicide-based alloy without RM additions [13] and (iv) the improved adhesion of the scale that formed at 1200 °C on the alloy Nb-12Ti-18Si-6Ta-2.5W-1Hf-5Sn-5Ge (alloy JZ2 in [14]).

The structure of the paper is as follows. In the next section, the raison d’être and the objectives of the research are discussed first, followed by the alloy design approach and its constraints and then by the selection of the alloys. The experimental details are then followed by the presentation of the results separately for the microstructures of the alloys and their isothermal oxidation at 800 and 1200 °C. The discussion considers the densities of the alloys, the macrosegregation of Si, their as cast and heat-treated microstructures and their oxidation. Then the experimental results are compared with the calculations (predictions) of NICE [4].

## 2. Alloy Design and Selection

### 2.1. Rationale and Objectives

Nowadays, Nb-silicide-based alloys can meet one or two of the three property goals [3] but to date there is no alloy that meets the toughness goal (minimum fracture toughness of 20 MPa√m for critical components [3,15]) and creep goal (see below), and simultaneously has the oxidation resistance required by the oxidation goal. The latter is a recession rate of less than 0.25 μm/h at 1315 °C. This goal is derived from the requirement of achieving the oxidation life at 1315 °C of the 2nd generation single crystal Ni-based superalloys at 1150 °C [15,16]. The toughness goal has been set because it is desirable that the new metallic materials should show some degree of metallic behaviour to distinguish them from engineering ceramic or ultra-high temperature ceramic candidate materials [17,18]. The toughness goal requires that the new metallic materials contain at least a small volume fraction of a ductile, metallic phase, which in the case of Nb-silicide-based alloys is the bcc Nb solid solution [15].

Nb-silicide-based alloys with transition metal (TM)/refractory metal (RM) additions meet (are close to) the creep property goal [4,5]. The latter expects the creep strength to be greater than 170 MPa at a creep rate of 2 × 10^−8^ s^−1^ at 1200 °C [3,15] (the creep goal assumes alloy density of 7 g/cm^3^, which can be met by Nb-silicide-based alloys [5]). Creep resistant alloys occupy a particular area in the Δχ versus δ or VEC versus δ maps, see area B in the Figure 1 in [19] and Figure 19 in [5] (for the parameters Δχ, δ and VEC see list of abbreviations and Appendix A). For example, polycrystalline Nb-silicide-based alloys with nominal compositions (at.%) Nb-18Si-xRM-5Hf and Nb-zTi-18Si-xRM-yHf (RM = Mo, Ta, W, x = 2, 3, 5, y = 1, 5 and z = 8, 11) lie in the area B in the Figure 1 in [19], and at 1200 °C and 200 MPa have creep rates (compressive creep) in the range 1.1 × 10^−6^ to 1.1 × 10^−8^ s^−1^, compared with creep rates in the range 1 × 10^−6^ to 4.4 × 10^−8^ s^−1^ that are predicted by NICE for T = 1200 °C and σ = 170 MPa. For the same conditions, the experimental creep rate of the single crystal CMSX-4 Ni-based superalloy is 5.6 × 10^−5^ s^−1^. To our knowledge, the alloy CMSX-4 is not used under the above conditions. The Ti/Hf and Nb/(Ti+Hf) ratios in Nb-silicide-based alloys are important for creep as are the addition of Al and Cr and their concentration [4,5,20]. For example, the creep of the MASC alloy (MASC = metal and silicide composite, nominal composition (at.%) Nb-25Ti-16Si-8Hf-2Al-2Cr [3,16]) deteriorated when Al and Cr were added to the “base” alloy Nb-25Ti-16Si-8Hf [5,6,20] and the creep of MASC-type alloys with/without RM additions deteriorated further at T > 1050 °C when the concentration of Al increased, compared with the MASC alloy and with Nb-silicide-based alloys with RM additions but without Al and Cr [5]. RHEAs and RCCAs are developed for structural applications for which creep is a key property, yet to date there is no data about the creep of these metallic materials [5].

Nb-silicide-based alloys, which have closed the gap with the oxidation goal significantly, have TM, simple metal and metalloid element additions, in particular additions of Al, B, Cr, Ge, Hf, Sn or Ti [4,5], and of these alloys those with B addition occupy a separate area in the Δχ versus δ or VEC versus δ maps compared with the creep resistant alloys (Figure 1 in [19]). Nb-silicide-based alloys with additions of Sn or Ge can be found in all areas in the aforementioned maps [5,19]. To date the effect of B, Ge or Sn addition on the properties of RHEAs and RCCAs has not been studied [2,5].

In Nb-silicide-based alloys, the Nb_ss_ is the Achilles heel for oxidation and creep owing to the poor oxidation of Nb and its sensitivity to interstitial contamination [4,5,21] and the inferior creep of Nb_ss_ compared with the silicides and other compounds that can be present in the microstructures of these alloys [4]. Nb-silicide-based alloys with Al and Cr additions can meet the toughness goal [3,5,16,22,23]. Furthermore, even though simple metal and metalloid element additions can supress pest oxidation and scale spallation at intermediate temperatures [4,8,9,10,11,12,24,25], avoidance of scale spallation is possible at high temperatures only when B is in synergy with Al, Cr and with Hf or Mo or Sn addition [5], or Al and Cr are present simultaneously with Ge and Sn in the alloy [13].

For Nb-silicide-based alloys with simultaneous addition of Ge and Sn with TMs, RMs, Al and Cr there is luck of data about their (i) microstructure, (ii) oxidation and (iii) creep. The (i) to (iii) were objectives of this research. The Nb solid solution can be Ti-rich Nb_ss_ or Si-free Nb_ss_ [26]. The latter is stable only when W or Mo and W are present without Al, Cr, Ge or Sn addition in the alloy [27,28]. Furthermore, Ti-rich Nb_ss_ tends to be poor in W and vice versa [28]. There is luck of data (iv) about the partitioning of Ti, Ta and W in the Nb_ss_ and (v) about the type(s) of Nb_ss_ that is(are) stable in Nb-silicide-based alloys with simultaneous addition of RMs, Sn, Ge, Al and Cr. The (iv) and (v) were objectives of this research.

Other research objectives were to compare the predicted by NICE [4] (vi) creep rate for the actual composition of the alloys JZ3 and JZ3+ (see Section 2.2.2) with the target one (see next section), and (vii) composition and vol.% of the Nb_ss_ in each alloy with the experimental data. Linked with the objective (ii) was the comparison of (viii) the calculated and experimental weight gains at 800 and 1200 °C (see next section) and (ix) the oxidation of the two alloys with the oxidation of RCCAs [5]. Another objective was (x) to compare the measured and predicted by NICE [4] macrosegregation of Si (MACSi) in each alloy. Regarding objective (x), we already knew (1) that the simultaneous addition of Sn with Al and Cr increased MACSi significantly [10,24], (2) that the synergy of Sn and Ge with Al and Cr also increased MACSi [13] and (3) that of the two elements Al and Cr, it is probably the former that plays a key role in Si macrosegregation (Tables 4–6 in [13]).

### 2.2. Alloy Design

We were interested in Nb-silicide-based alloys of the Nb-Ti-Si-Ta-W-Hf-Ge-Sn-Al-Cr system and used the alloy design methodology NICE [4] to design/select alloy(s). The reasons for the inclusion of the aforementioned elements in the alloys to be studied were outlined in the Section 1 and Section 2.1 and were discussed in [4,5,13,14,19,26]. We expected difficulties in making alloys with composition close to nominal (see Section 2.2.2), owing to us using arc melting and alloying elements with a very wide range of melting temperatures (T_m_^Sn^ = 232 °C, T_m_^Al^ = 660 °C, T_m_^Ge^ = 937 °C, T_m_^Si^ = 1412 °C, T_m_^Ti^ = 1667 °C, T_m_^Cr^ = 1857 °C, T_m_^Hf+^ = 2227 °C, T_m_^Nb^ = 2467 °C, T_m_^Ta^ = 2980 °C and T_m_^W^ = 3400 °C) and our experience from the research described in [14].

The starting points of alloy design were the target (1) for creep rate έ = 1 × 10^−7^ s^−1^ at T = 1200 °C and σ = 170 MPa, for the reasons discussed in [4], and (2) for the weight changes after 100 h isothermal oxidation at 800 and 1200 °C, respectively of 10 and 50 mg/cm^2^. Alloy selection was assisted from data about how the alloying additions of Al, Cr, Ge and Sn affect the macrosegregation of Si in RM free alloys (Tables 4–6 in [13]). The creep target was the same as that used for the design of the two alloys that were studied in [14]. The oxidation target was considered realistic for Nb-silicide-based alloys (see [5] and the Section 2.4 in [4]) and was set bearing in mind the results about the oxidation of the alloy JZ2 [14].

#### 2.2.1. Constraints of Alloy Design

The alloy composition was selected using NICE following the procedure described in [4] and with the following specific constraints about alloying additions. The alloy should

(a) lie in the domain defined by the areas A and B in the Δχ versus δ map of Nb-silicide-based alloys [5,19],
(b) contain Al and Cr (because of objectives (i), (ii) and (iii)),
(c) have density lower than state of the art Ni-based superalloys (ρ ≈ 9 g/cm^3^ for 3rd generation, ρ ≈ 8.64 to 8.95 g/cm^3^ for 2nd generation [3,29]), lower than the density of single phase bcc solid solution RCCAs with Al, Nb and Ta additions (ρ ≈ 6.85 to 9.08 g/cm^3^ [2]), and multiphase bcc solid solution + intermetallic(s) (Laves, M_5_Si_3_) RCCAs with Al, Nb and Ta or Cr, Nb and Ta additions (ρ ≈ 7.14 to 8.58 g/cm^3^ [2]). To our knowledge there is no data about the density of RCCAs with simultaneous Al, Cr, Nb and Ta additions, and there is no data about RCCAs with Ge and Sn addition [2],
(d) contain Ta and W with Ta/W = 2.4, which is higher than the ratio in RCCAs studied to date (Ta/W ≤ 1 [2]) and to have low ductile to brittle transition temperature (DBTT), owing to the strong negative effect of W on DBTT compared with Ta [5,30],
(e) contain Ti and Hf with Ti/Hf = 12, which is higher than the ratio in RCCAs studied to date (Ti/Hf ≤ 3 [2]) and the ratio suggested in [16] (see also [5]) and to have low DBTT [30],
(f) have Al/Cr = 1 and Sn/Ge = 1 based on the results reported in [13] and
(g) have Ge and Sn concentrations each of 5 at.% (based on the results reported in [10,11,13]).

As regards the above constraints, the (a), (d) and (e) were related to the creep target and data about creep in [4,6], the constraints (b), (d), (e) and (g) were linked with (c), and the constraints (b), (f) and (g) were linked with oxidation resistance. The choice of the concentrations of Al, Cr, Ge and Sn, and thus constraints (f) and (g), were informed by recent literature about the effect of the synergy of Al and Cr with Sn or Ge on the oxidation behaviour of Nb-silicide-based alloys [4,7,8,9,10,11,24,31].

#### 2.2.2. Alloy Selection

The nominal composition of the selected alloy was Nb-12Ti-18Si-6Ta-2.5W-1Hf-5Sn-5Ge-5Al-5Cr. We shall call this alloy JZ3. As we shall discuss in the next section, we encountered difficulties with the control of the Sn/Ge ratio owing to losses by evaporation during arc melting. To compensate for this loss we increased the Sn concentration to 7.5 at.%, and thus we also report in this paper our results for a second alloy, which we shall call JZ3+ (the plus sign is used to indicate the increase in the concentration of Sn, which was not prescribed by NICE but by alloy making difficulties). In other words, the nominal composition of JZ3+ was Nb-12Ti-18Si-6Ta-2.5W-1Hf-7.5Sn-5Ge-5Al-5Cr. Both alloys could be considered to be RCCAs, according to the “definition” of the latter in [2] (see also [5]).

## 3. Experimental

Buttons (25 g) of both alloys were produced using high purity elements (better than 99.99 wt.%) and arc melting with a non-consumable tungsten electrode. Melting was done in a water-cooled copper crucible and in an argon atmosphere. Each alloy was melted five times to ensure as much as possible chemical homogeneity. The melt solidified in the water-cooled copper crucible. Similarly, with the research described in [14], we found it very difficult to make an alloy with composition as close as possible to the nominal one, owing to loss by evaporation of elements with low melting points. In the same way as in [14], we made up for the elemental losses by slightly increasing the weight of lost elements. After many attempts, the “best” two alloys were selected for further study (see Section 2.2.2).

The alloy buttons were sectioned to produce specimens to characterise their as cast microstructures in different parts of the buttons in order to find out if there were differences in the microstructures that solidified under different cooling rates. According to DSC experiments (data not shown) no melting occurred in each alloy up to 1600 °C. The specimens that were used for heat treatment were from the bulk of the buttons. Each specimen was wrapped in Ta foil. The heat treatment was done at 1500 °C for 100 h in a tube furnace under a flow of Ti gettered argon. Each specimen was furnace cooled. For microstructural characterization the specimens were mounted in Bakelite, ground using 120, 400, 800 and 1200 grit papers, and polished to 1 μm surface finish.

A NETZSCH STA 449 F3 thermal analyser was used for the study of the isothermal oxidation of the alloys at 800 °C and 1200 °C. The oxidation specimens (3 × 3 × 3 mm^3^, ground to 1200 grit) were cut from the cast buttons. For the thermogravimetric (TG) studies the heating and cooling rate was 3 °C per minute. The oxidised samples were cold mounted in resin and polished. An AccuPyc II 1340 gas pycnometer was used to measure the density of the alloys.

We used X-ray diffraction (XRD, Siemens D5000 X-ray diffractometer with monochromatic CuKα radiation, HiltonBrooks Ltd., Crew, UK), scanning electron microscopy (Inspect F SEM, JEOL 6400 SEM (JEOL Ltd., Tokyo, Japan) and Philips XL 30S FEG SEM instruments (ThermoFisher Scientific, Hillsboro, OR, USA)) and energy dispersive X-ray spectrometry (EDS) to characterize the microstructures. Phases were identified using the Powder Diffraction File data and the ICDD PDF-4+ (International Centre Diffraction Data, Newton Square, PA, USA) and Sieve+ software. Chemical compositions of large areas from the top, bulk and bottom of the buttons and of constituent phases with size larger than 5 μm were analysed in JEOL 6400 SEM and Philips XL 30S FEG SEM instruments under a voltage of 20 kV. For the EDS, specimens of high purity Nb, Ti, Si, Hf, Ta, W, Ge, Sn, Al, Cr and Al_2_O_3_ that were polished to 1 μm finish were used as standards. Calibration of the EDS detector was done every hour during analysis using a specimen of pure Co. At least 5 EDS analyses of large areas or phases were done. Area fractions of Nb_ss_ were calculated using the software Image-Pro and BSE images. In the tables, the chemical analysis data is given with the average, minimum and maximum values and standard deviation.

## 4. Results

### 4.1. Microstructures

The densities of the as cast alloys and the vol.% of the Nb_ss_ and A15-Nb_3_X (X = Al, Ge, Si, Sn) in the as cast and heat treated alloys are given in the Table 1. The density of the alloy JZ3 was higher than that of the alloy JZ3+, as was the case for the vol.% of the aforementioned phases. The average chemical compositions of the alloys and a summary of the phases in their microstructures is given in the Table 2. The chemical analysis data for the as cast (AC) and heat treated (HT) alloys is given in the Tables S1 to S4 in the Supplemental data. In JZ3-AC the concentrations of Sn and Si, respectively were lower and very close to the nominal ones. In [JZ3+]-AC the Si concentration was higher than the nominal and the ratio Sn/Ge = 1 required from the alloy design (constraint f, Section 2.2.1) was slightly exceeded. There was macrosegregation of Si (MACSi) in both alloys, respectively 4 and 3.1 at.%.

**Figure 1:**
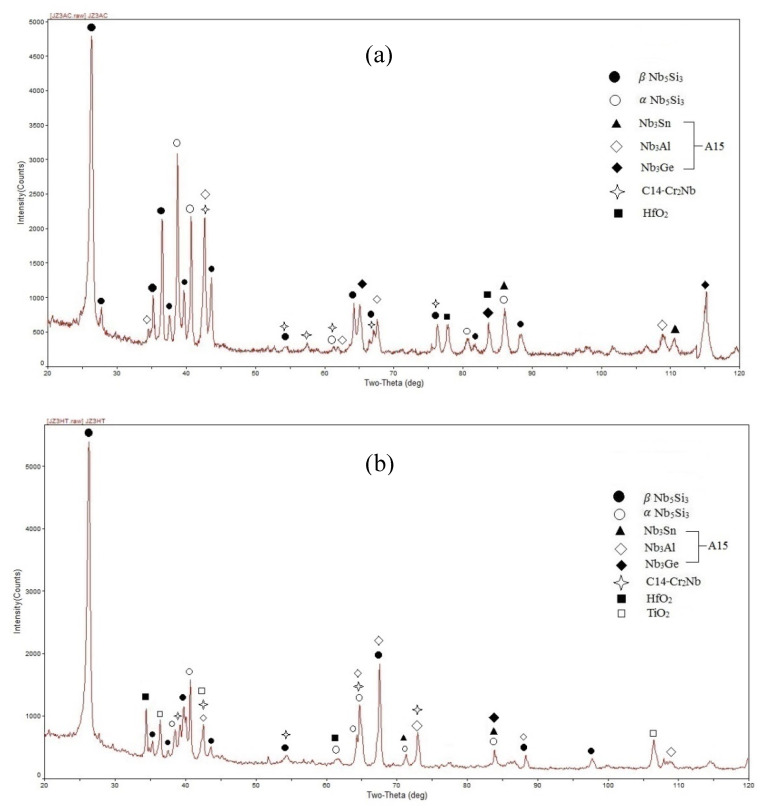
The X-ray diffractograms of the alloy JZ3. (**a**) AC and (**b**) HT.

**Figure 2:**
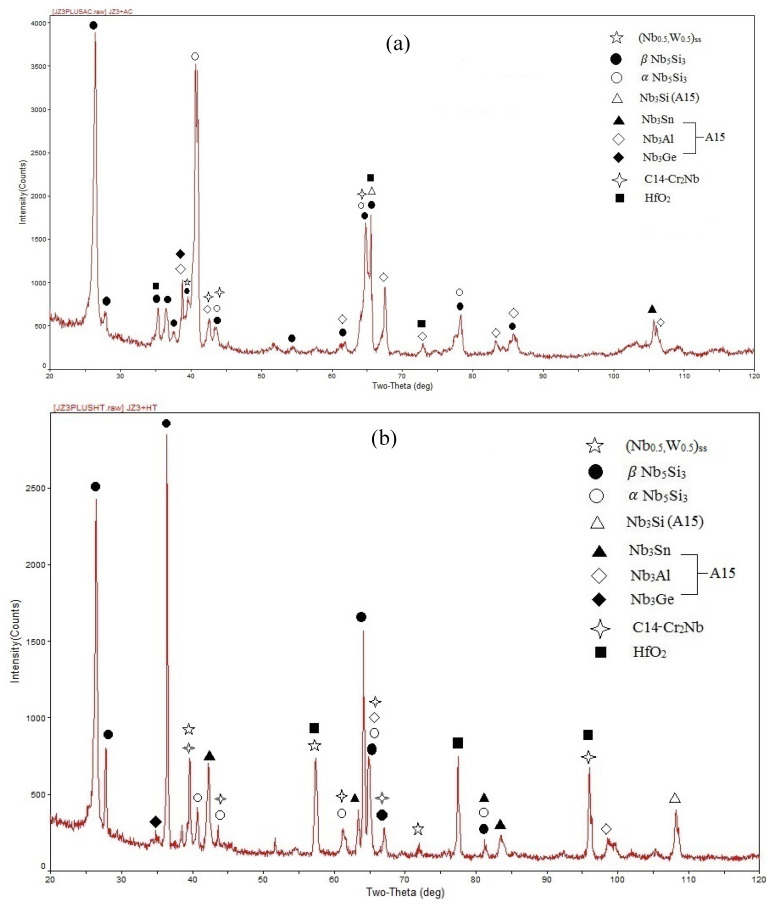
The X-ray diffractograms of the alloy JZ3+. (**a**) AC and (**b**) HT.

According to the XRD (Figure 1 and Figure 2) and EDS data (Tables S1–S4) the same phases were present in both the alloys, namely the solid solution and Nb_5_Si_3_, A15-Nb_3_X, C14-Cr_2_Nb Laves and HfO_2_. In JZ3 the Nb_ss_ was only confirmed by the EDS analysis (Tables S1 and S2). In JZ3+ the solid solution was rich in W and is indicated as (Nb,W)_ss_ in Figure 2 and Tables ST3 and Tables ST4. Both the β and αNb_5_Si_3_ were present in the cast and heat-treated microstructures of the alloys (Figure 1 and Figure 2) as well as Ti-rich Nb_5_Si_3_ (Table 2, and Tables S1 to S4). Owing to partitioning of solutes, the A15-Nb_3_X was also Ti or Cr-rich in JZ3-AC (Table S1) and Ti-rich in [JZ3+]-AC (Table S3). In both alloys the A15-Nb_3_X was homogenised after the heat treatment (Tables S2 and S4).

**Figure 3:**
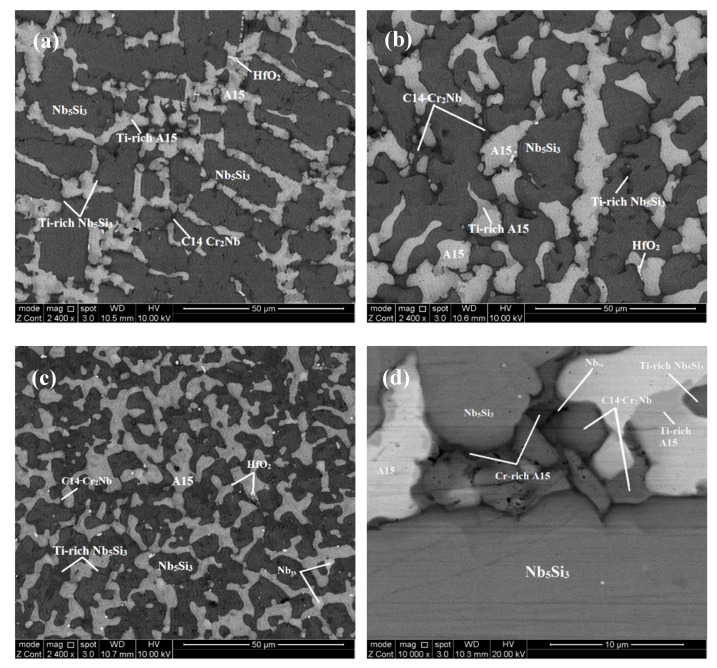
BSE images of the microstructure of the alloy JZ3-AC in the (**a**) top, (**b**) bulk, (**c**) bottom. Details of bulk microstructure are shown in (**d**).

The microstructure in the top and bulk of the button of JZ3-AC (Figure 3a,b) consisted of the primary Nb_5_Si_3_, and the A15-Nb_3_X, Nb_ss_ and Laves phases. The Nb_5_Si_3_ and Laves phases exhibited similar contrast under BSE imaging. Ti-rich areas were observed in the Nb_5_Si_3_ and A15-Nb_3_X. The concentrations of Al and Cr in the Nb_5_Si_3_ were in agreement with previous research [25,32] and the Si content was decreased due to its substitution by Al, Ge and Sn [32]. The Si+Sn+Ge+Al content in the Nb_5_Si_3_ and Ti-rich Nb_5_Si_3_ was 38.7 at.% and 36.6 at.%, respectively. The Ta+W content in the A15 and Ti-rich A15 was 16.3 at.% and 12.1 at.%, respectively. Cr-rich A15-Nb_3_X was observed in the bulk of the button near the C14-Cr_2_Nb Laves phase. The Cr-rich A15 was also rich in Ti, Si and Al but lean in Ta and W compared with the “normal” and Ti-rich A15, see Figure 3d and Table S1. The Si+Sn+Ge+Al content in the Cr-rich A15 was 24.4%, higher than that in the normal and Ti-rich A15 (19.4 at.%). Only a very small volume fraction of the solid solution was formed in the top and bulk that was rich in Ti, Al and Cr. The Laves phase was poor in Sn and Ge. In the bottom of the button the Nb_ss_ was rich in Ta, W and Cr and lean in Sn and Ge. The volume fraction of the Laves phase in the bottom of the button was lower than those in the top and bulk. In JZ3-AC the vol.% of the A15-Nb_3_X compound was about 27% (Table 1).

**Figure 4:**
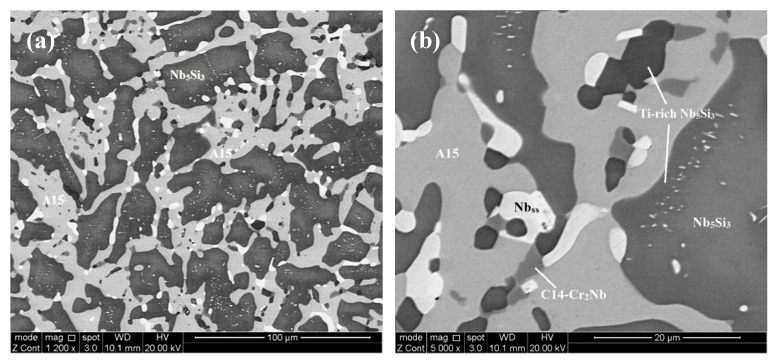
BSE images of the bulk microstructure of the alloy JZ3-HT: (**a**) low and (**b**) higher magnification images showing precipitation of second phase in silicide grains.

There was chemical inhomogeneity of Si in JZ3-HT after the heat treatment. The microstructure had coarsened and consisted of Nb_5_Si_3_ grains surrounded by A15-Nb_3_X, Nb_ss_ that exhibited white contrast, and C14-Cr_2_Nb and HfO_2_, see Figure 4 and the Figure 1b. Ti rich areas were observed only in the Nb_5_Si_3_ and the enrichment in Ti was relatively more severe in some small Nb_5_Si_3_ grains adjacent to the Laves phase. No Ti rich areas were found in the A15 phase. There were many tiny particles with contrast similar to that of the Nb_ss_ that were dispersed in Nb_5_Si_3_ (Figure 4b). There was no noticeable change in the average composition of the Nb_5_Si_3_ after the heat treatment (Tables S1 and S2). The Ti, Al and Cr concentrations in the A15-Nb_3_X had increased and the Ta and W concentrations decreased. The Si+Sn+Ge+Al and Ta+W contents in the A15 were 20.6 at.% and 12.4 at.%, respectively. The significant increase of the concentration of W to 17 at.% and the reductions of the Sn and Al contents by 1.1 at.% and 2.1 at.%, respectively in the Nb_ss_ should be noted. The Si content in the Laves phase was increased by 2.4 at.% and the Ti content was reduced by 4.8 at.%. The Nb_ss_, A15 and Laves phases were essentially Hf free due to the formation of HfO_2_. The areas near the surface of the specimen were contaminated by oxygen. In these areas, Ti oxide formed (Figure 1b) that exhibited black contrast. The vol.% of the A15 phase had increased (Table 1).

**Figure 5:**
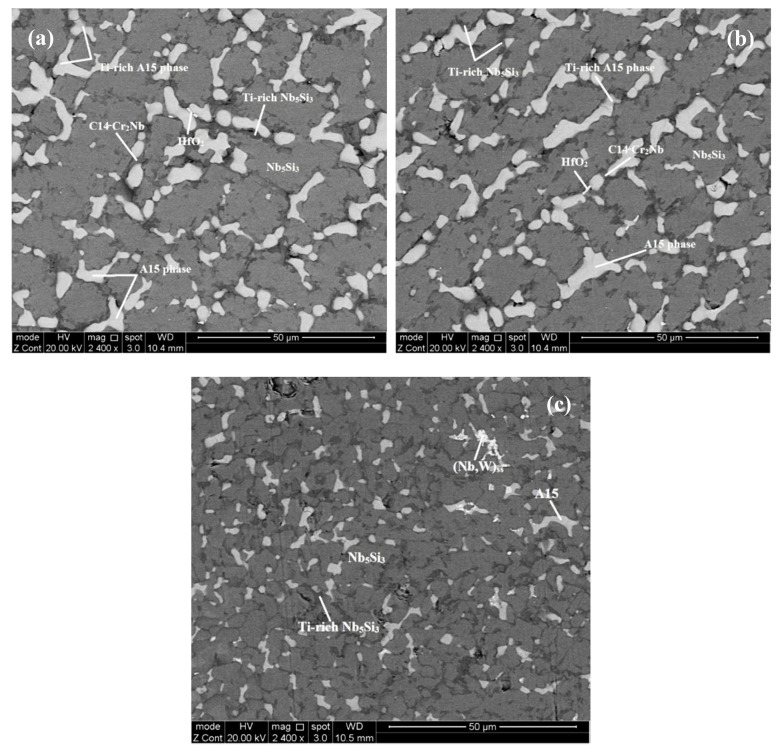
BSE images of the microstructure of the alloy [JZ3+]-AC in the (**a**) top, (**b**) bulk and (**c**) bottom.

The microstructure in the top and bulk of [JZ3+]-AC was slightly coarser compared with JZ3-AC (Figure 5a,b), and consisted of the A15-Nb_3_X and C14-Cr_2_Nb Laves phases that were formed between grains of primary Nb_5_Si_3_. The volume fraction of the A15 was lower than that in JZ3-AC (Table 1). The Si+Sn+Ge+Al content in the normal and Ti-rich Nb_5_Si_3_ was 39.3 at.% and 37.3 at.%, respectively. The Sn contents in the normal and Ti-rich A15 were increased, and the solubility of Si, Ge, Ta and W was decreased, compared with JZ3-AC. The Si+Sn+Ge+Al content in the normal and Ti-rich A15 was 21.8 at.% and 22.7 at.%, respectively and the Ta+W content was 15.2 at.% and 8.9 at.%, respectively. Compared with JZ3-AC, there was no significant change in the composition of the Laves phase, with the exception of small changes in the concentrations of Ti and Al. The microstructure of the bottom of the button was finer (Figure 5c) where the (Nb,W)_ss_ and HfO_2_ exhibited white contrast. The solubility of Ta in the (Nb,W)_ss_ was high (15.2 at.%) and the Si content was low (1.7 at.%), see Table S3, compared with the Nb_ss_ in the as cast alloys JZ1 and JZ2 [14] and JZ3-AC.

**Figure 6:**
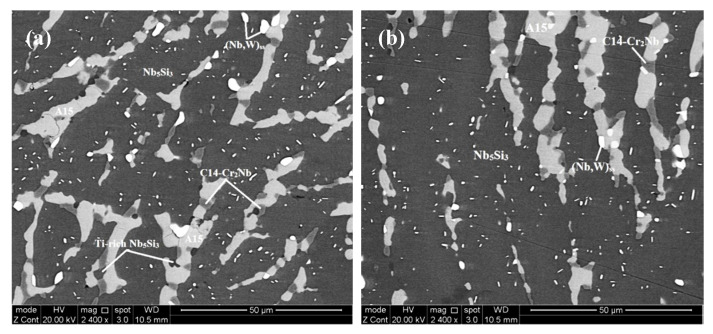
BSE images of the microstructure of the heat-treated alloy JZ3+ (**a**) in the bulk and (**b**) below the surface.

In [JZ3+]-HT, there was chemical inhomogeneity of Si. Compared with [JZ3+]-AC, the Sn/Ge ratio was reduced to 0.94 from 1.16 and the Al/Cr ratio had increased to 0.99 from 0.88, in other words these ratios had shifted closer to those required by alloy design (constraint f, Section 2.2.1). The heat-treated microstructure is shown in Figure 6. No Ti rich areas were observed in the large Nb_5_Si_3_ grains but adjacent to the Laves phase some small Nb_5_Si_3_ grains were rich in Ti, the same as in JZ3-HT. The W rich (Nb,W)_ss_ was observed adjacent to large Nb_5_Si_3_ grains. Two analyses of the solid solution corresponded to Si-free (Nb,W)_ss_ with composition 31.2Nb-4.5Ti-0Si-15.3Ta-37.4W-0-Hf-0.9Sn-0.1Ge-1.4Al-9.1Cr, i.e., slightly richer in Cr and poorer in W compared with the (Nb,W)_ss_ in Table S4. Tiny precipitates with contrast similar to that of the (Nb,W)_ss_ were dispersed in large Nb_5_Si_3_ grains (Figure 6). The volume fractions of the A15 and solid solution were lower than those in JZ3-HT, see Table 1. In the Nb_5_Si_3_, there were changes in the Ti, Ta, W and Cr concentrations, the Si content decreased to 23.8 at.% and the contents of Sn and Al increased (Table S4). In the Ti-rich Nb_5_Si_3_ the Si and Hf content increased and the Sn content decreased. The Si+Sn+Ge+Al contents in the normal and Ti-rich Nb_5_Si_3_ were the same (≈ 38.3 at.%). The Si+Sn+Ge+Al and Ta+W contents in the A15 were 20.4 at.% and 14.7 at.%, respectively. The W concentration in the (Nb,W)_ss_ was increased by 4.5 at.% and some grains of Si-free Nb_ss_ had formed. No contamination by oxygen was observed below the surface of [JZ3+]-HT.

### 4.2. Oxidation

#### 4.2.1. Oxidation at 800 °C

**Figure 7:**
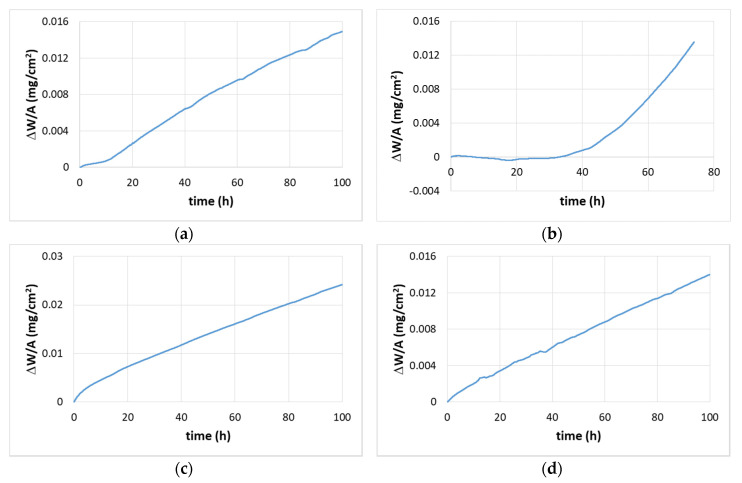
Thermogravimetric (TG) data; (**a**,**b**) for 800 °C and (**c**,**d**) for 1200 °C, (**a**,**c**) alloy JZ3 and (**b**,**d**) alloy JZ3+.

**Figure 8:**
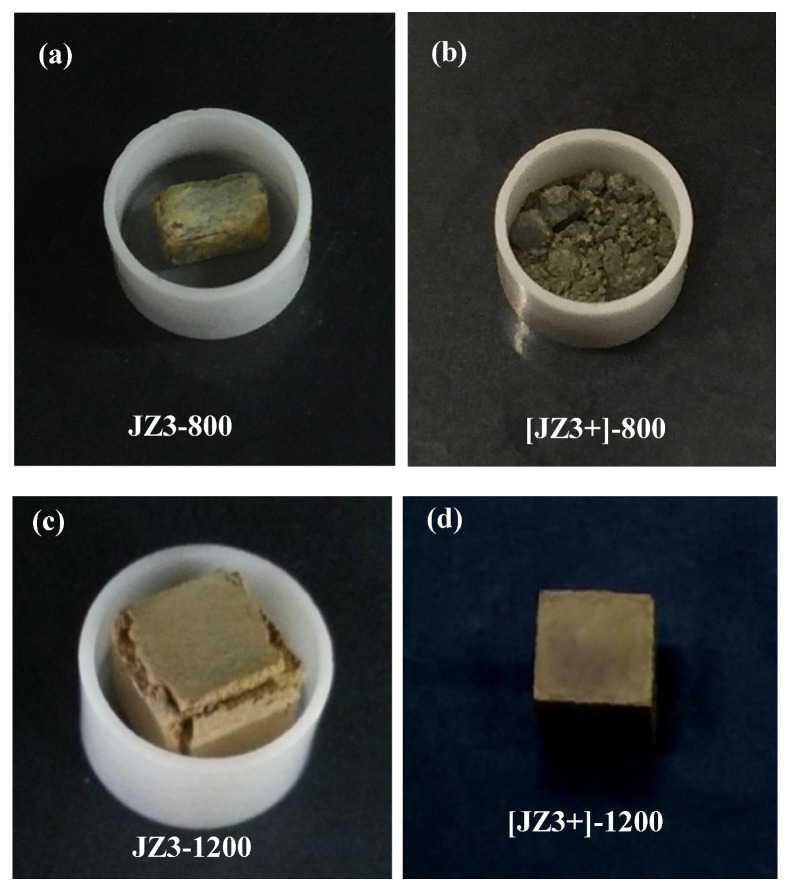
The specimens of the alloys JZ3 (**a**,**c**) and JZ3+ (**b**,**d**) after isothermal oxidation at 800 °C (**a**,**b**) and 1200 °C (**c**,**d**).

The TG data of the alloys is shown in the Figure 7a,b and the specimens after the oxidation experiments are shown in Figure 8a,b. The alloy JZ3+ suffered from catastrophic pest oxidation. The weight changes of the alloys JZ3 and JZ3+, respectively were 14.9 mg/cm^2^ after 100 h and 13.9 mg/cm^2^ after 74 h (Table 3). The oxidation of both alloys followed linear kinetics (Table 3). The weight change of JZ3 increased steadily after about 8 h. The weight change of the alloy JZ3+ was minimal up to about 40 h, with small weight loss between 7 and 33 h, and increased dramatically after this time, leading to catastrophic oxidation after 74 h. No pest oxidation and good adherence of the oxide scale were observed in the alloy JZ3, which, compared with the oxidation of JZ1 and JZ2 [14], would suggest that Al and Cr improved the properties of the scale at 800 °C. Owing to the pesting of the alloy JZ3+, only the microstructure of the alloy JZ3 was studied after the isothermal oxidation at 800 °C.

**Figure 9:**
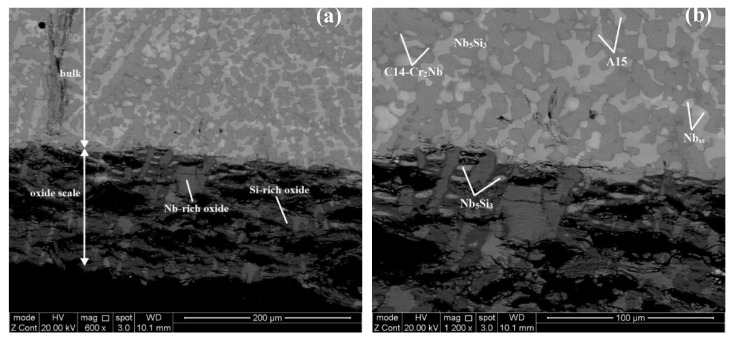
BSE images of the microstructure of a cross section of the oxidized alloy JZ3 at 800 °C: (**a**) low and (**b**) higher magnification images showing details of oxide scale and of the microstructure of the substrate below the scale.

A cross section of the alloy JZ3 is shown in Figure 9. The bottom layer in Figure 9a that exhibits darker contrast is the oxide scale. The latter was severely cracked and the cracks were parallel to the surface. The thickness of the scale was about 150 μm. A diffusion zone was not observed. The chemical analysis data of the phases in the oxide scale and bulk is given in the Table S5 in the Supplemental data. The scale consisted of Nb-rich and Si-rich oxides exhibiting bright and dark contrast, respectively. There were remnants of Nb_5_Si_3_ in the scale, with composition essentially the same as that of the silicide in the bulk, which indicated that the oxidation proceeded with the inward diffusion of oxygen. The phases in the bulk were Nb_5_Si_3_, A15-Nb_3_X, Nb_ss_ and C14-Cr_2_Nb Laves with Ti-rich Nb_5_Si_3_ silicide and Ti-rich A15. The Cr-rich A15 that was observed in JZ3-AC was not present. There was no noticeable changes in the compositions of the Nb_5_Si_3_, A15 and Laves phases compared with JZ3-AC. Compared with the starting microstructure in the specimen prior to oxidation, the Ti-rich Nb_5_Si_3_ was leaner in Ti, the Ti-rich A15 leaner in Ti and Cr and the Nb_ss_ leaner in Ti and Al, and richer in Ta and W. Furthermore, the Ti concentration in the Ti-rich A15 and the Nb_ss_ was close to the compositions of the A15 and Nb_ss_ in the heat-treated alloy. The same was the case for the Ta content of the Nb_ss_.

#### 4.2.2. Oxidation at 1200 °C

**Figure 10:**
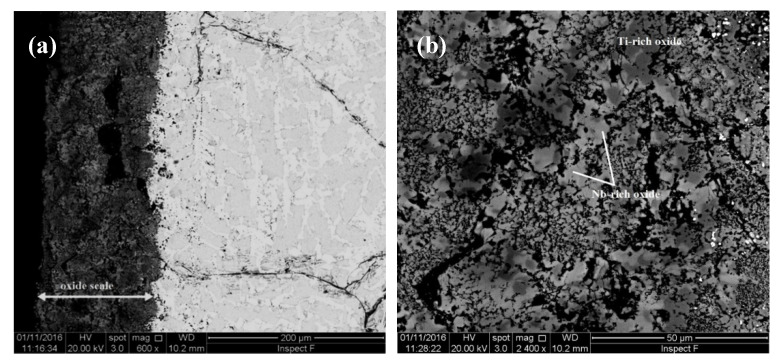

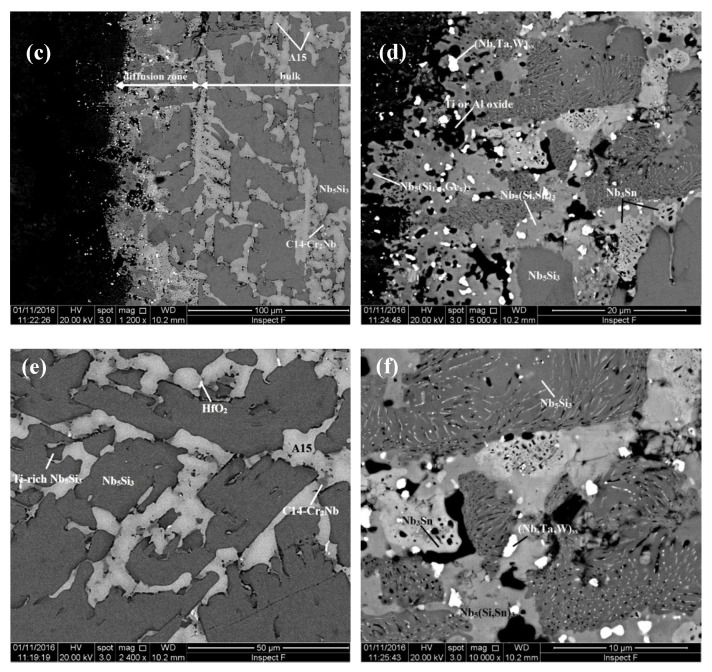
BSE images of the microstructure of a cross section of the oxidized alloy JZ3 at 1200 °C, (**a**,**b**) show the microstructure of the oxide scale, (**c**) diffusion zone and bulk, (**d**) micro-structure of the diffusion zone, (**e**) microstructure of the bulk and (**f**) white-line feature in the Nb_5_Si_3_ (see text).

**Figure 11:**
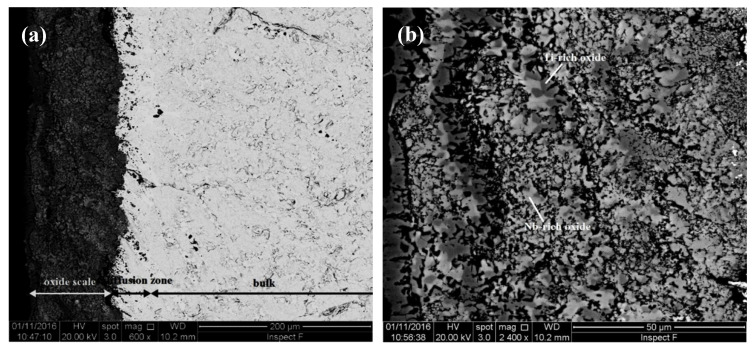

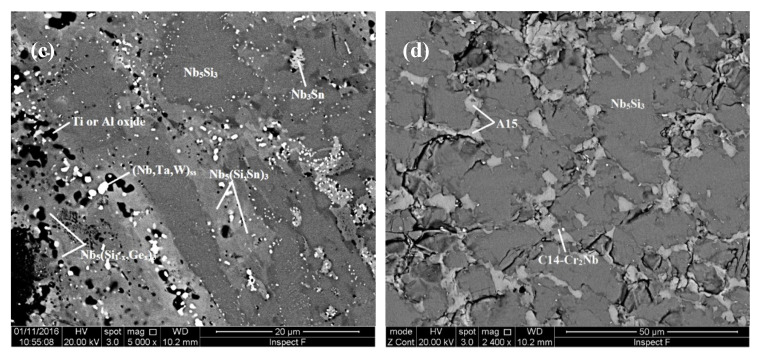
BSE images of the microstructure of a cross section of the oxidized alloy JZ3+ at 1200 °C, (**a**,**b**) show the microstructure of the oxide scale, (**c**) microstructure of the diffusion zone and (**d**) bulk microstructure.

The TG data is shown in the Figure 7c,d and the weight changes and oxidation rate constants are given in the Table 3. The weight gains of JZ3 and JZ3+ were 24.21 mg/cm^2^ and 14.02 mg/cm^2^, respectively. The alloy JZ3 followed parabolic oxidation kinetics for the first 9 h of oxidation and then linear kinetics. The alloy JZ3+ followed parabolic oxidation throughout the experiment with lower rate for the first 14 h of oxidation. The adhesion of the scale on JZ3 improved compared with JZ2 [14] and remarkably there was no scale spallation in JZ3+ (Figure 8d). The microstructures of cross sections of the oxidised specimens of the alloys JZ3 and JZ3+ are shown respectively in the Figure 10 and Figure 11. In these Figures we can see details of the oxide scale, diffusion zone and bulk.

The composition of the phases in the different regions of the oxidised alloy JZ3 are given in the Table S6 in the Supplemental data. Nb-rich and Ti-rich oxides were formed in the oxide scale. Silicon rich oxide was also present and exhibited black contrast, the same as the pores and cracks in the scale. Hafnia was not observed in the scale. All three oxides were free of Sn and Ge. The Ti-rich oxide was richer in Al and Cr compared with the Nb-rich oxide. The Nb_5_(Si,Sn)_3_, Nb_5_(Si_1-x_,Ge_x_)_3_, Nb_3_Sn and the Ta and W rich solid solution (Nb,W)_ss_ with W/Ta ≈ 3.2 were observed in the diffusion zone. The former and latter 5-3 silicide were present in the inner and outer regions of the diffusion zone, respectively. Nb_5_Si_3_ contaminated by oxygen was also present in the diffusion zone and contained needles of hafnia. With the exception of Nb_5_Si_3_, the other phases in the diffusion zone were all very lean in Ti and Al owing to the consumption of these elements in the formation of the scale. A small volume fraction of HfO_2_ was present in the diffusion zone. The hafnia exhibited the same contrast as the solid solution. Some of the black contrast phases just below the scale were Ti or Al oxide(s). All phases in the diffusion zone were contaminated by oxygen, the silicides with up to about 4 at.% oxygen and the A15 and solid solution with about 6 at.% oxygen. The bulk had similar microstructure to that of JZ3-AC but no solid solution was observed. Compared with JZ3-AC, the Ti-rich Nb_5_Si_3_ was leaner in Ti, the A15 leaner in Si and the Laves phase leaner in Ti and Al, and richer in Ta and Cr. Compared with JZ3-HT, the Ti-rich Nb_5_Si_3_ was leaner in Ti and richer in Si, the A15 leaner in Si, the Laves phase leaner in Hf and the normal Nb_5_Si_3_ leaner in Si. All the phases in the bulk were not contaminated by oxygen.

The oxide scale that formed on the alloy JZ3+ consisted of Nb-rich, Ti-rich and Si-rich oxides (Table S7 in the Supplemental data). The Nb_5_Si_3,_ Nb_5_(Si,Sn)_3_, Nb_5_(Si_1-x_,Ge_x_)_3_, A15-Nb_3_X compounds, the (Nb,W)_ss_ with W/Ta ≈ 3.6 and HfO_2_, Ti and Al oxides were observed in the diffusion zone. The Ti and Al oxides were observed only at the scale/diffusion zone interface. The composition of the A15 compound in the diffusion zone is not given in the Table S7 because many small particles of the solid solution were dispersed in it. Tin and Ge were not present in the scale. The Ti-rich oxide was richer in Al and Cr compared with the Nb-rich oxide. The solubility of Al and Ti in the Nb_5_(Si,Sn)_3_ did not change with position in the diffusion zone. The Nb_5_(Si_1-x_,Ge_x_)_3_ formed in the areas just below the oxide scale and was lean in Ti and Al owing to the consumption of these elements to form Ti and/or Al oxides in these areas. The same was the case for the solid solution which was very rich in W and Ta. All the phases in the diffusion zone were contaminated by oxygen but their contamination was less severe compared with JZ3. The bulk microstructure consisted of Nb_5_Si_3_, A15-Nb_3_X, C14-Cr_2_Nb and hafnia. Similarly with JZ3, there was no solid solution in the bulk of the oxidised JZ3+ (Table S7 and Figure 11). The composition of the Nb_5_Si_3_ in the bulk did not change after the oxidation. However, some Nb_5_Si_3_ grains were severely cracked. This cracking of the Nb_5_Si_3_ in the bulk was not observed in the alloys JZ2 and JZ3. Compared with [JZ3+]-AC, the normal Nb_5_Si_3_ was leaner in Sn, the Ti-rich Nb_5_Si_3_ and A15 leaner in Si and the Laves phase leaner in Ti and richer in Si and Ta.

## 5. Discussion

### 5.1. Density

The densities of both alloys (Table 1) were lower than those of the alloys JZ1 and JZ2 [14]. Considering the constraint (c) in alloy design (Section 2.2.1), the densities of both alloys were lower than the densities of Ni-based superalloys. Compared with the data for Nb-silicide-based alloys with RM, TM additions with/out Al [5], the density of the alloy JZ3 was lower than that of Nb-10Ti-18Si-5Hf-5Mo-3W (ρ = 8.39 g/cm^3^, έ = 5.1 × 10^−7^ s^−1^) and the density of the alloy JZ3+ was lower than that of Nb-10Ti-18Si-5Al-5Hf-5Mo-3W (ρ = 7.82 g/cm^3^, έ = 1.1 × 10^−6^ s^−1^), where the creep rates are for the creep goal conditions [5]. In addition, the densities were lower than the target density (ρ = 9 g/cm^3^) of Nb-silicide-based alloys with RM additions and with strength of 450 MPa at 1500 °C [33].

#### Comparison with RCCAs

The alloys JZ3 and JZ3+ could be considered to be RCCAs [2], see Section 2.2.2. The density of both alloys was lower than the upper density value of (a) transition metal HEA (9 g/cm^3^ [2]), (b) single phase bcc solid solution RCCAs with Al, Nb and Ta additions (9.08 g/cm^3^ [2]) and (c) multiphase bcc solid solution + intermetallic(s) (Laves, M_5_Si_3_) RCCAs with Al, Nb and Ta or Cr, Nb and Ta additions (8.58 g/cm^3^ [2]).

### 5.2. Macrosegregation

The macrosegregation of an element i (MACi) in Nb-silicide-based alloys has been defined as MACi = C_max_^i^ − C_min_^i^ [34]. Macrosegregation is present when MACi > 2 at.%. MACi in Nb-silicide-based alloys is linked with solute partitioning [34,35]. Tin and Ge have a strong effect on MACSi [10,35]. The latter increases when the parameters ΔH_m_/T_m_ (“alloy entropy of fusion”), T_m_^sp^ (melting temperature of sp electronic configuration elements) and [ΔH_m_/T_m_] × [ΔH_m_^sd^/ΔH_m_^sp^]^−1^ increase, and the parameters—ΔH_m_^sd^/ΔH_m_^sp^, T_m_^sd^/T_m_^sp^, ΔH_m_ (“alloy enthalpy of melting”), T_m_ (alloy melting temperature) and T_m_^sd^ (melting temperature of the sd electronic configuration elements) decrease [34].

There was macrosegregation of Si in the alloys JZ3 and JZ3+ with MACSi values of 4 at.% and 3.1 at.%, respectively. Compared with the alloy OHS1 (Nb-24Ti-18Si-5Al-5Cr-5Ge-5Sn [13]) where MACSi = 6.8 at.%, the simultaneous addition of Al and Cr with Ge, Sn, Ta and W, reduced the macro-segregation of Si. We shall use the data for the alloy JZ3 to discuss the aforementioned trends and the effect of the Al and Cr addition.

Table 4 shows (i) that the said trends are followed by the parameters ΔH_m_/T_m_, ΔH_m_^sd^/ΔH_m_^sp^, T_m_^sd^, T_m_^sp^, T_m_^sd^/T_m_^sp^ and [ΔH_m_/T_m_] × [ΔH_m_^sd^/ΔH_m_^sp^]^−1^ and that the simultaneous addition of Hf, Ta and W in the alloy JZ3 (compared with OHS1) did not affect the same trends that are obeyed also by the alloys KZ5, KZ6 [36] and OHS1. It should be noted that the above mentioned trend for T_m_ is followed by the alloys KZ6, JZ3 and OHS1. The data in the Table 4 for the alloys KZ5 and KZ6 were calculated using the actual compositions of the cast alloys given in [36].

In the Table 5 the alloys KZ7 and ZF5 are compared with the alloy JZ3 in order to consider the Al effect on MACSi. The parameters were calculated using the actual compositions of the cast alloys in [36,37]. Table 5 shows (i) that the simultaneous addition of Hf, Sn, Ta and W in JZ3 increased MACSi and (ii) that the aforementioned trends were followed by the parameters ΔH_m_/T_m_, ΔH_m_^sd^/ΔH_m_^sp^, T_m_^sp^, T_m_^sd^/T_m_^sp^ and [ΔH_m_/T_m_] × [ΔH_m_^sd^/ΔH_m_^sp^]^−1^ with the exception of the parameters T_m_^sd^, ΔH_m_ and T_m_. However, when the data for the alloy JZ3+ is compared with the data for the alloys ZF5 and KZ7 (table not shown), the same conclusion as (i) above is reached and (iii) ΔH_m_ is the only parameter for which the trend is not followed.

The effect of Cr on MACSi can be understood using the data in the Table 6. In this table the data for the alloys KZ4 and OHS1, which was given in the Table 6 in [13], is compared with the alloy JZ3. The data in Table 6 shows that the aforementioned trends are followed by the parameters T_m_, ΔH_m_/T_m_, ΔH_m_^sd^/ΔH_m_^sp^, T_m_^sd^, T_m_^sp^, T_m_^sd^/T_m_^sp^ and [ΔH_m_/T_m_] × [ΔH_m_^sd^/ΔH_m_^sp^]^−1^ with the exception only of the parameter ΔH_m_. However, when the data for the alloy JZ3+ is compared with the data for the same alloys (i.e., KZ4 and OHS1, table not shown), only the trends for the parameters T_m_, T_m_^sd^ and ΔH_m_^sd^/ΔH_m_^sp^ are followed.

The above data would suggest (a) that when Al and Cr are in synergy with Ge, Hf, Sn, Ta and W simultaneously (1) MACSi is reduced compared with the macrosegregation in the absence of Hf, Ta and W additions (alloy OHS1) and (2) their effect on MACSi is essentially the same, even though, as concluded in [13], (a) Al in synergy with Ge or Sn individually increases MACSi, (b) Sn has a stronger effect than Ge on MACSi and (c) the combined effect of Ge and Sn is enhanced when Al, Ge and Sn are present simultaneously in the alloy.

The important result of the research reported in this paper is the low macrosegregation of Si in two arc melted Nb-silicide-based alloys in which RM (important for strength and creep), TM and simple metal and metalloid elements (important for oxidation) were present simultaneously.

### 5.3. Microstructures

Compared with the alloy JZ2 (actual composition Nb-11.8Ti-21.5Si-5.2Ta-2W-2.8Sn-5.2Ge-1Hf [14]), the addition of Al and Cr and the increase in the concentrations of Ge and Sn in the alloy JZ3 resulted to the complete suppression of the formation of the Nb_ss_ + Nb_5_Si_3_ eutectic and a change in the “architecture” of the cast microstructure. Research on Ti-rich alloys based on Nb-24Ti-18Si-5Al-5Cr [38] (base alloy) with Ge (Nb-24Ti-18Si-5Al-5Cr-5Ge [35]) or Sn (Nb-24Ti-18Si-5Al-5Cr-5Sn [10]) or Ta (Nb-24Ti-18Si-6Ta-5Al-5Cr [36]) addition has shown that the formation of the Nb_ss_ + Nb_5_Si_3_ eutectic, which was not suppressed by the addition of Ta [36], was partially suppressed by Ge [35] and completely suppressed by Sn [10]. The architecture of the microstructure of JZ3-AC (Figure 3) was similar to that that resulted from the addition of Sn in Nb-24Ti-18Si-5Al-5Cr-5Sn [10]. The microstructure formed in the alloy JZ3 was attributed to (i) the increased Sn concentration in the alloy JZ3 (compared with the alloy JZ2 [14]) and (ii) the synergy of Sn with Al and Cr in JZ3 (the same was the case with the alloy Nb-24Ti-18Si-5Al-5Cr-5Sn [10]).

Owing to the higher concentration of Sn in the alloy JZ3, and the results reported in [10] and the data in NICE [4], the A15-Nb_3_X compound was expected to be stable in the microstructure. This was confirmed by the experimental results. In the alloy JZ3, as the primary βNb_5_Si_3_ formed the surrounding melt became richer in Al, Cr, Sn, Ti and W owing to the partitioning of these solutes. Between the Nb_5_Si_3_ dendrites, the A15, Laves and Nb_ss_ phases formed (Figure 3).

The solute additions Al, Ge, Si and Sn can form with Nb or Cr the A15 compounds Nb_3_Al, Nb_3_Ge, Nb_3_Si (metastable), Nb_3_Sn, Cr_3_Si and Cr_3_Ge [39]. The Ti_3_Sn is not an A15 compound (DO_19_ structure). Titanium however can substitute Nb in A15 compounds. Nb forms the C14-Cr_2_Nb Laves phase as do Hf, Ta and Ti that form HfCr_2_, TaCr_2_ and TiCr_2_ Laves phases, respectively [39]. If we consider the melting temperatures of the aforementioned A15 compounds, and of the Nb_ss_ in the top and bulk of JZ3-AC (Nb_ss_^b^ in Table S1) and that the phase with the highest melting temperature should form first, then their ranking is Nb_3_Sn (2130 °C), Nb_3_Al (2060 °C), Nb_3_Ge (1900 °C), Nb_ss_^b^ (1866 °C), Cr_2_Nb (1730 °C). Thus, in the melt surrounding the primary βNb_5_Si_3_ the A15 phase formed first followed by the Nb_ss_^b^ and then the C14-Cr_2_Nb Laves phase that formed in the last to solidify melt [38]. As the A15 compound formed the surrounding melt became richer in Ti, Si, Ge, Cr and poorer in Sn, Ta and W, and the Ti-rich A15 and Cr-rich A15 formed, which made the melt rich in Ge and Si, and poor in Ti, Al, Cr, Hf, Sn and then from this melt the Nb_ss_^b^ formed making the surrounding melt rich in Si, Ta, W, Ge and poor in Ti, Hf, Al, Cr. It is suggested that in the top and bulk of the alloy JZ3-AC the solidification path was L → L + βNb_5_Si_3_ → L + βNb_5_Si_3_ + A15 → L + βNb_5_Si_3_ + A15 + C14-Cr_2_Nb → βNb_5_Si_3_ + A15 + C14-Cr_2_Nb + Nb_ss_^b^. The formation of αNb_5_Si_3_ occurred from the solid state transformation βNb_5_Si_3_ → αNb_5_Si_3_.

In the bottom of the button of JZ3-AC the vol.% of the C14-Cr_2_Nb Laves phase was reduced and the solid solution was rich in Cr, Ta and W (Nb_ss_^c^ in Table S1). If we consider the melting temperatures of the aforementioned A15 compounds and Nb_ss_^c^, the ranking is Nb_ss_^c^ (2225 °C), Nb_3_Sn (2130 °C), Nb_3_Al (2060 °C), Nb_3_Ge (1900 °C), Cr_2_Nb (1730 °C). Thus, in the bottom of JZ3-AC in the melt surrounding the primary βNb_5_Si_3_ the Nb_ss_^c^ formed first, and then the A15 phase followed by the C14-Cr_2_Nb Laves phase that again formed in the last to solidify melt. As the Nb_ss_^c^ was rich in Cr, Ta and W (all three elements also are constituents of the NbCr_2_ Laves phase, see Table S1) a lower vol.% of the Laves phase formed in the last to solidify melt. The high concentrations of Cr, Ta and W in the Nb_ss_^c^ also affected the partitioning of solutes in the A15 and the formation of the Ti-rich and Cr-rich A15. It is suggested that in the bottom of JZ3-AC the solidification path was L → L + βNb_5_Si_3_ → L + βNb_5_Si_3_ + Nb_ss_^c^ → L + βNb_5_Si_3_ + Nb_ss_^c^ + A15 → L + βNb_5_Si_3_ + Nb_ss_^c^ + A15 + C14-Cr_2_Nb.

The architecture of the microstructure of [JZ3+]-AC was similar to that of JZ3-AC. Formation of A15-Nb_3_X in JZ3+ “was assured” by its higher Sn content. In the top and bulk of the button, as the primary βNb_5_Si_3_ formed_,_ the surrounding melt became rich in Ti, W, Sn, Al and Cr. Considering the melting temperature of the phases, in the top and bulk of the button, in the melt that surrounded the βNb_5_Si_3_ silicide the A15 compound formed first followed by the C14-Cr_2_Nb. In these parts of [JZ3+]-AC it is suggested that the solidification path was L → L + βNb_5_Si_3_ → L + βNb_5_Si_3_ + A15 → βNb_5_Si_3_ + A15 + C14-Cr_2_Nb. In the bottom of the button the partitioning of solute elements made the melt surrounding the primary βNb_5_Si_3_ silicide rich in W (see below). From this W rich melt formed the W rich solid solution. The melting temperature of the (Nb,W)_ss_ that formed in the bottom of the button of JZ3+ was estimated to be about 2300 °C, higher than those of the A15 and Laves phases. It is suggested that in the bottom of [JZ3+]-AC the solidification path was L → L + βNb_5_Si_3_ → L + βNb_5_Si_3_ + (Nb,W)_ss_ → L + βNb_5_Si_3_ + (Nb,W)_ss_ + A15 → βNb_5_Si_3_ + (Nb,W)_ss_ + A15 + C14-Cr_2_Nb.

**Figure 12:**
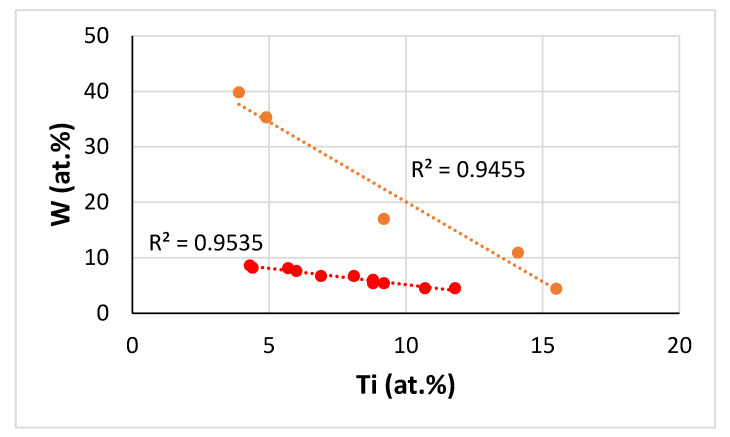
Tungsten versus Ti concentration in Nb_ss_ in the alloys JZ2, JZ3 and JZ3+ (orange data) and Nb-silicide-based alloys with Hf, Mo, Si, Ti and W additions [28,40].

The chemical composition of the top, bulk and bottom of the cast button was (within experimental error) not different in each alloy (Tables S1 and S3). In the alloy JZ3+ the concentration of Sn was increased to 5.7 at.% for the reasons discussed in the Section 2.2.2. The cast microstructure of JZ3+ was very similar to that of the alloy JZ3-AC with the only difference that no solid solution formed in the top and bulk of the button of JZ3+. Solid solution was observed only in the bottom of the button of JZ3+. The XRD and EDS data respectively suggested that the solid solution in JZ3+ was (Nb,W)_ss_ (Figure 2a) and very rich in W and poor in Ti (Table S3). The EDS data about the solid solutions that formed in the alloys JZ1, JZ2 [14], JZ3 and JZ3+ is consistent with other data about Nb-silicide-based alloys that shows that “Ti and W do not like each other in the Nb_ss_” [28]. Figure 12 compares data for the W and Ti contents in solid solutions in Nb-silicide-based alloys with RM and Ti addition but without Ge and/or Sn addition and the data for the alloys JZ2, JZ3 and JZ3+. Both sets of data in Figure 12 confirm that as the Ti concentration in the solid solution increases the W content decreases, in agreement with [28], and also show that when W and Ti are in synergy with Ge and Sn “the dislike between W and Ti becomes stronger”.

Comparison of the data for the solid solutions in the alloys JZ1, JZ2 [14] JZ3 and JZ3+ would suggest (i) that the solubility of the refractory metals in the Nb_ss_ increased as a result of their synergy with Al and Cr, (ii) that the solubility of both refractory metals, in particular W, in the Nb_ss_ increased with increased concentration of Sn in the alloy and (iii) that solidification conditions near the water cooled copper crucible were important regarding the type of solid solution that formed in the alloys JZ3 and JZ3+.

Tantalum and W-rich solid solution existed only in the bottom of the buttons of the alloys JZ3-AC and [JZ3+]-AC with Ta/W ratio of 0.95 (for Nb_ss_^c^) and 0.43 (for (Nb,W)_ss_) in each alloy, respectively. In these two alloys the Nb_ss_^c^ (Table S1) and (Nb,W)_ss_ (Table S3) formed in the melt that surrounded the primary βNb_5_Si_3_. What can we deduce about the composition of the melt?

The data for the normal Nb_5_Si_3_ in the alloys JZ1, JZ2 [14], JZ3 and JZ3+ show that the “partitioning ratio” X_Nb5Si3_/X_alloy_ of each element X, where X = Ti, Si, Ta, W, Sn, Ge, Hf, Al, Cr, was not significantly different among the alloys (average partitioning ratio values for each one of the aforementioned elements, respectively 0.72, 1.57, 0.92, 0.4, 0.36, 1.25, 0.6, 0.5, 0.2). The data for the partitioning ratio X_Nb5Si3_/X_alloy_ for the Ti-rich Nb_5_Si_3_ in the alloys JZ1, JZ2 and JZ3 were not very different for X = Ti, Si, Sn, Ge, Hf with average values 1.1, 1.4, 0.6, 1.5, 1.2, respectively. For the Ti-rich Nb_5_Si_3_ in JZ3+ the partitioning ratio X_Nb5Si3_/X_alloy_ was higher for Ti, Sn, Hf, Al (1.73, 1.42, 2, 1.6, respectively), lower for Si and Ge (0.85, 1.1, respectively) and for Ta and W (0.6, 0.17, respectively) lower than the average value for the normal Nb_5_Si_3_. The partitioning ratio of Cr in the Ti-rich Nb_5_Si_3_ in JZ3 and JZ3+ was higher (0.68) than that in the normal Nb_5_Si_3_ (0.25). Thus, in JZ3+, after the Ti-rich Nb_5_Si_3_ formed in the bottom of the button, the surrounding melt became richer in Si, Ta, W, Ge and poorer in Ti, Sn, Hf, Al and Cr, compared with the same melt in the alloys JZ1, JZ2 and JZ3 (Al and Cr only in JZ3). The solid solution formed from this melt and had lower Ti, Sn, Hf, Al, Cr and higher Ta and W than the solid solution in JZ3, in agreement with the experimental results (Table 2 and Table 4).

Tungsten is the strongest solid solution strengthener of Nb compared with other RMs [4,5,21,30,41], thus it is anticipated that the hardness of (Nb,W)_ss_ in JZ3+ was significantly higher than that of the solid solution in JZ3. The hardness of the Nb_ss_^c^ and (Nb,W)_ss_, respectively in the alloys JZ3 and JZ3+ could not be measured owing to their small size and low volume fraction.

Let us now turn our attention to the Nb_5_Si_3_ silicide and A15 compound in the alloys JZ3 and JZ3+, whose chemical compositions were given in the Tables S1 and S3. The Al and Cr concentrations in the Nb_5_Si_3_ were increased in the Ti-rich regions of the silicide, in agreement with previous research. Unlike the alloys JZ1-AC and JZ2-AC [14] and JZ3-AC, the concentration of Si in the Ti-rich Nb_5_Si_3_ of [JZ3+]-AC was low (16.7 at.%) and the concentrations of Sn and Al were significantly higher (respectively 8.1 at.% and 7.3 at.% in JZ3+ compared with 2 and 4.6 at.% in JZ3). However, in both alloys the Al+Ge+Si+Sn content in the Ti-rich Nb_5_Si_3_ was essentially the same (40.1 at.% in JZ3 and 41 at.% in JZ3+) and consistent with the Si content of βNb_5_Si_3_ in the Nb-Si binary [42].

**Figure 13:**
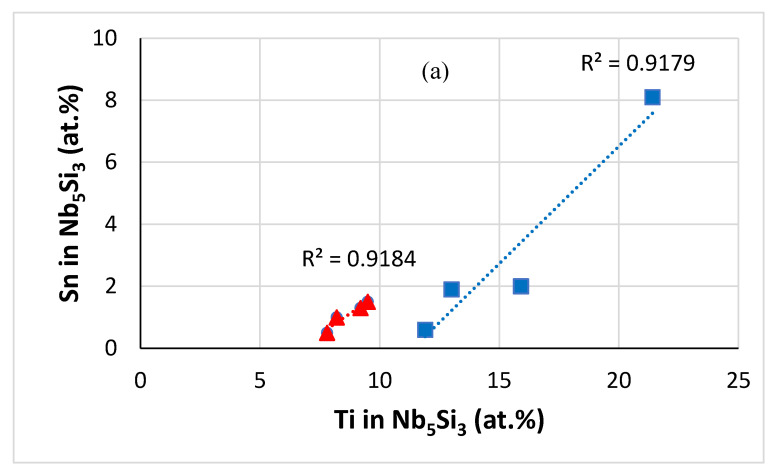

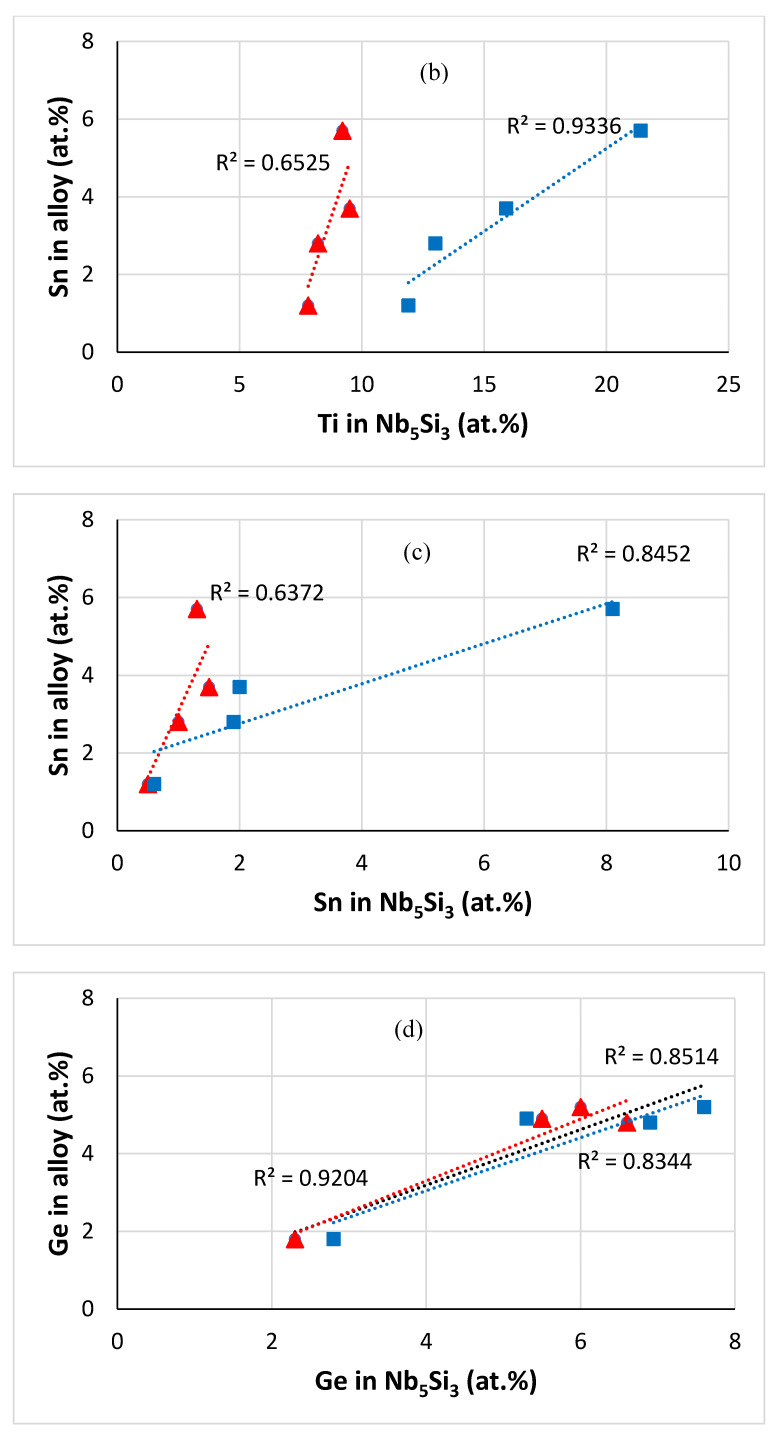

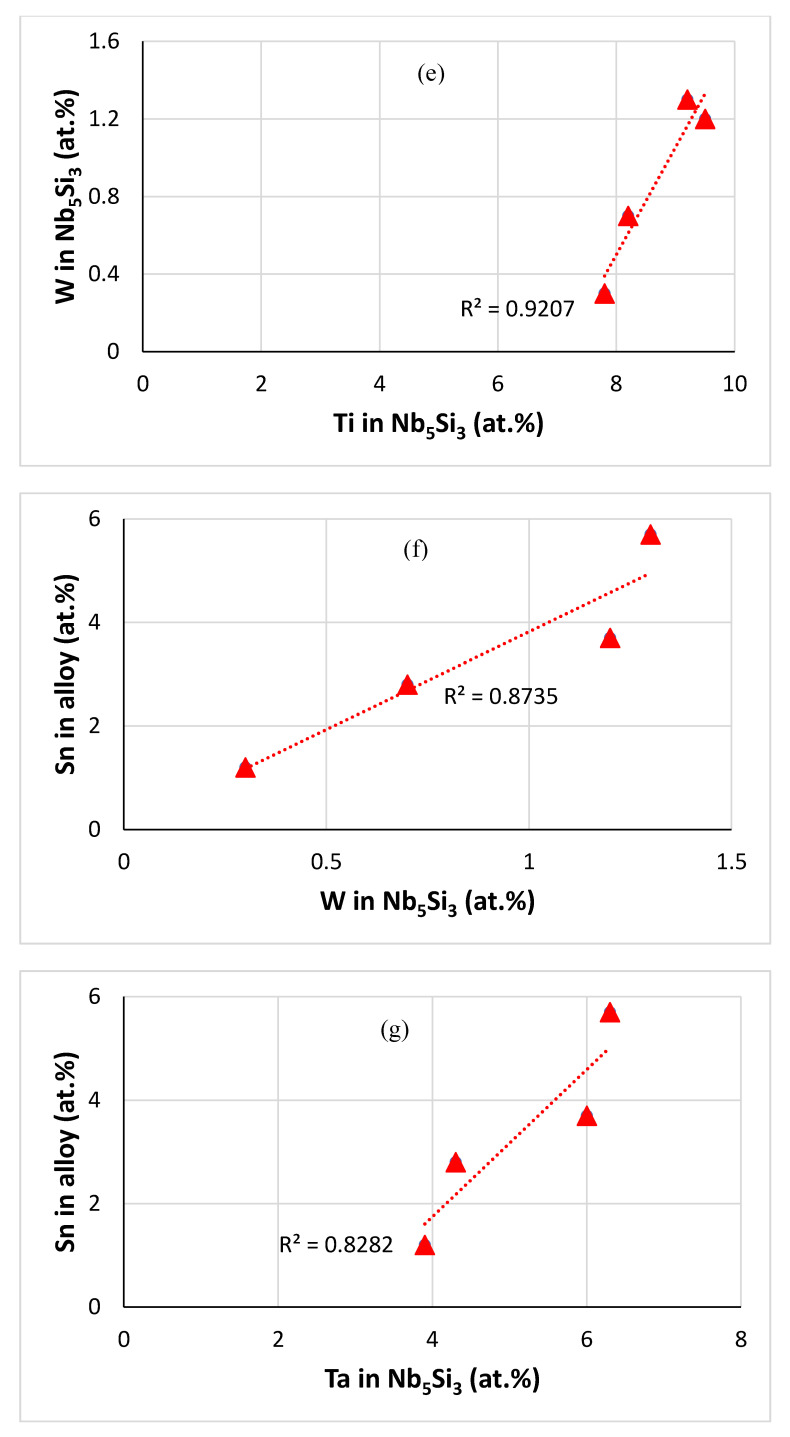
(**a**) Sn in Nb_5_Si_3_ versus Ti in Nb_5_Si_3_, (**b**) Sn in the alloy versus Ti in Nb_5_Si_3_, (**c**) Sn in alloy versus Sn in Nb_5_Si_3_, (**d**) Ge in alloy versus Ge in Nb_5_Si_3_, (**e**) W in Nb_5_Si_3_ versus Ti in Nb_5_Si_3_, (**f**) Sn in the alloy versus W in Nb_5_Si_3_, (**g**) Sn in the alloy versus Ta in Nb_5_Si_3_, blue squares for Ti-rich Nb_5_Si_3_, red triangles for normal Nb_5_Si_3_. In (**d**) R^2^ = 0.8514 and R^2^ = 0.9204 for linear fit, respectively of all data and for normal Nb_5_Si_3_. Data for the alloys JZ1, JZ2 [14], JZ3 and JZ3+.

In Nb-silicide-based alloys the concentration of Sn in the Nb_5_Si_3_ increases with increasing Ti content in the silicide (Figure 1 in [32]). Figure 13 shows (a) that the same trend was followed when Sn was in synergy with RM, TM and Ge in the alloys JZ1, JZ2 [14], JZ3 and JZ3+ regardless of the presence or not of Al and Cr in the alloy (Figure 13a,b), (b) that the slopes of the data for Sn in the normal and Ti-rich Nb_5_Si_3_ essentially were the same (Figure 13a,c) that the data sets for Sn in the normal and Ti-rich Nb_5_Si_3_ were separate (Figure 13a). Furthermore, Figure 13b,c shows (d) that the Ti or Sn concentration in the normal and Ti-rich Nb_5_Si_3_ increased with increasing Sn concentration in the alloy with the ratio (element X in Nb_5_Si_3_/Sn in alloy) higher for the Ti-rich Nb_5_Si_3_ (X = Sn, Ti) and (e) that the Ge concentration in the normal and Ti-rich Nb_5_Si_3_ increased with increasing Ge concentration in the alloy (Figure 13d), with the ratio (Ge in Nb_5_Si_3_/Ge in alloy) essentially the same for the normal and Ti-rich Nb_5_Si_3_. The data for Ti and W in the normal Nb_5_Si_3_ are shown in Figure 13e–g. The concentration of W in the normal Nb_5_Si_3_ increases with the Ti content of the silicide (Figure 13e) and the same is the case for Ta (figure not shown). Furthermore, the concentration of Ta and W in Nb_5_Si_3_ increases as the Sn content in the alloy increases (Figure 13f,g). Note that trends similar to those shown in Figure 13e–g, which are for the normal Nb_5_Si_3_, were not followed (meaning the R^2^ values were low) in Ti-rich Nb_5_Si_3_.

Compared with the hardness of unalloyed Nb_5_Si_3_, the hardness of the silicide (a) decreases when the latter is alloyed with (1) Ti, (2) Sn and Ti, (3) Ti and Al, (4) Ti and Cr, (5) Ti, Al and Cr, (6) Ti, Sn and Cr, (7) Hf and Al, (8) Hf and Sn, (9) Ti, Hf and Sn, (10) Ti, Hf, Al and Sn but (b) increases when the silicide is alloyed with (11) Ge, (12) Ge and Ti, (13) Ge and Al, (14) Ti, Al and Ge, (15) Ti, Cr and Ge [32]. The (Nb,Ti,Cr)_5_(Si,Ge)_3_ exhibits the highest hardness. The hardness of the silicide in the alloys JZ3 and JZ3+, respectively was 1712 ± 40 and 1826 ± 54 HV.

**Figure 14:**
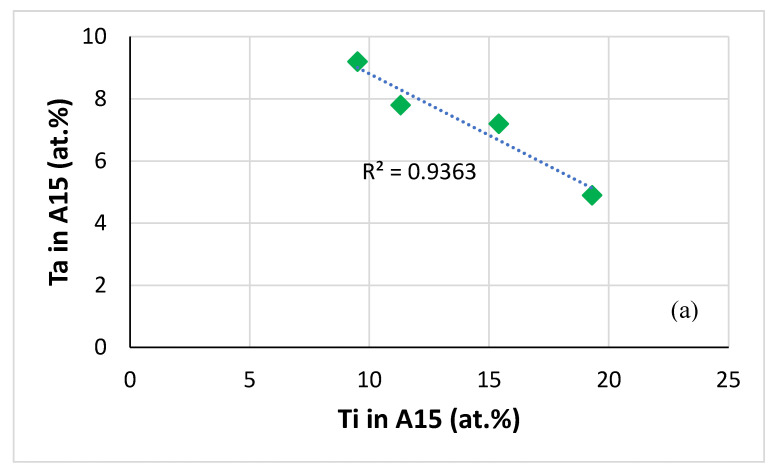

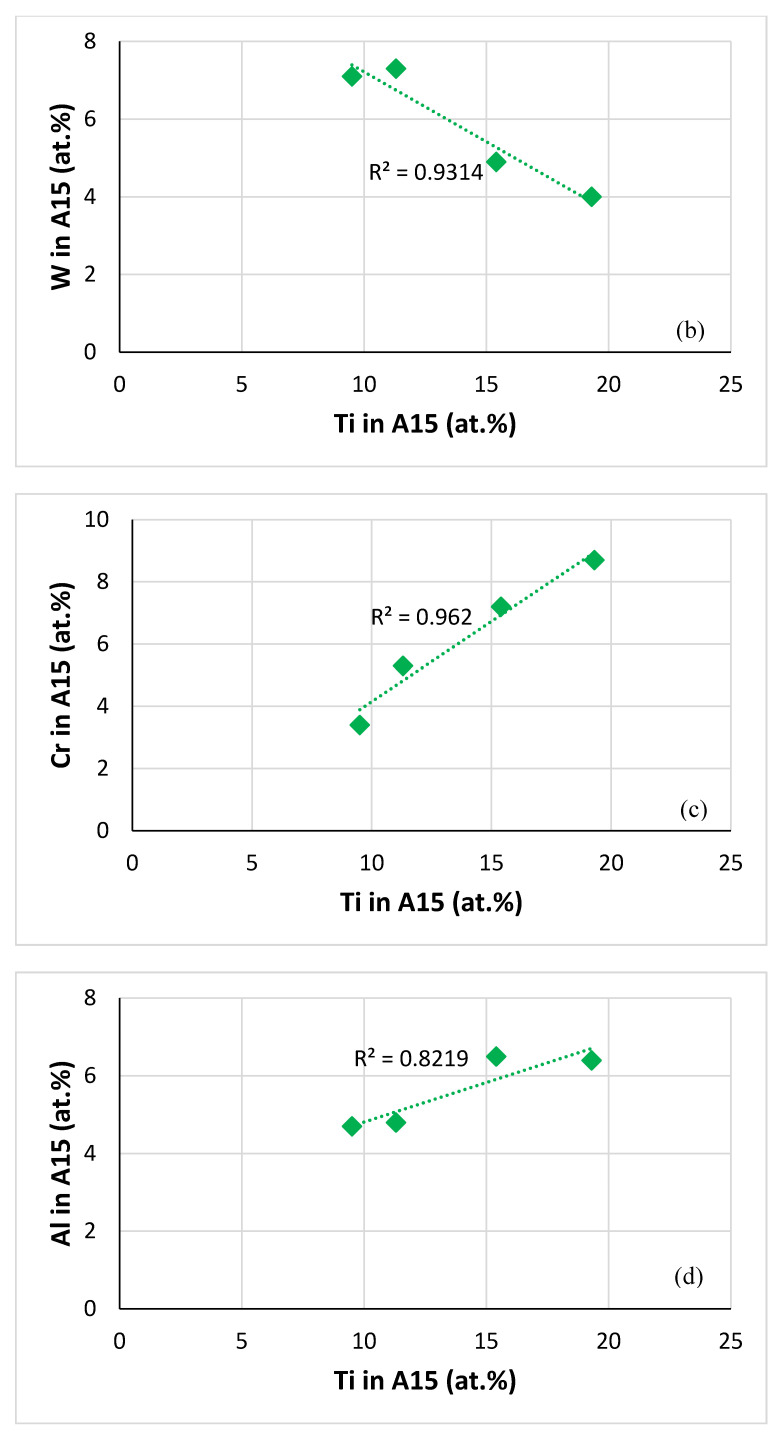
Data for the interdependence of Ti and solute concentrations in A15-Nb_3_X in the alloys JZ3 and JZ3+. (**a**) Ta versus Ti, (**b**) W versus Ti, (**c**) Cr versus Ti and (**d**) Al versus Ti.

Data for the A15 compound is shown in Figure 14. The concentrations of Ta and W decrease with increasing Ti concentration (the same is the case for Ge, Figure not shown), which is the opposite trend than that exhibited by Ta and W in normal Nb_5_Si_3_ (see above), whereas the concentrations of Al and Cr in A15 increase with increasing Ti concentration (the same is the case for Sn, figure not shown). The Al+Ge+Si+Sn content of the A15 in both alloys was within the range of X in A15-Nb_3_X (X = Al, Ge, Si, Sn) [39,42,43,44]. The Cr+Al+Ge+Si+Sn content of the C14-Laves phase increased slightly after the heat treatment and was within the range of values reported for the NbCr_2_ Laves phase in Nb-silicide-based alloys [45]. For both alloys the values of the parameters VEC and Δχ of the A15 and Laves phases were within the ranges reported for Nb-silicide-based alloys [45], VEC = 5.179, Δχ = −0.359 and VEC = 5.186, Δχ = −0.3762 for the Laves phase, respectively in the cast alloys JZ3 and JZ3+, and VEC = 4.727, Δχ = 0.916 and VEC = 4.747, Δχ = 0.925 for the A15, respectively in the heat-treated alloys JZ3 and JZ3+. Furthermore, for the Laves phase the ratio R_<Nb>_/R_<Cr>_ was 0.78 and 0.74, respectively for the cast alloys JZ3 and JZ3+, in agreement with [45].

In the microstructures of the heat-treated alloys the W-rich solid solution, the C14-Cr_2_Nb Laves and A15 phases and β and αNb_5_Si_3_ were present with Ti rich regions only in the 5-3 silicide. These were the stable phases in both alloys. In the solid solution the Ta/W ratio in JZ3 and JZ3+ was reduced, respectively to 0.73 and 0.38, compared with the cast alloys, consistent with the increased W concentration in each solid solution after the heat treatment. The VEC parameter of the A15 compound was outside the range 4.63 to 4.72, in agreement with [45]. It should be noted that in alloys without Ge, Sn and refractory metals, the concentration of Cr should be ≥ 8 at% in order for the C14-Cr_2_Nb to be stable in the microstructure [36,38]. The presence of both β and αNb_5_Si_3_ in the heat treated alloys (as was the case for the cast alloys) was attributed to the effects of alloying additions (Ge,Ta,W,Sn) on the transformation βNb_5_Si_3_ → αNb_5_Si_3_, as discussed for the alloys JZ1 and JZ2 in [14] and the effects of Al and Cr on the stability of βNb_5_Si_3_ [36,38]. The precipitation of a phase with contrast similar to that of the solid solution that was observed in Nb_5_Si_3_ grains of both the heat treated alloys (Figure 4 and Figure 6) is in agreement with previous research and was attributed to the transformation of βNb_5_Si_3_ to αNb_5_Si_3_ during heat treatment [28,36,38].

**Figure 15:**
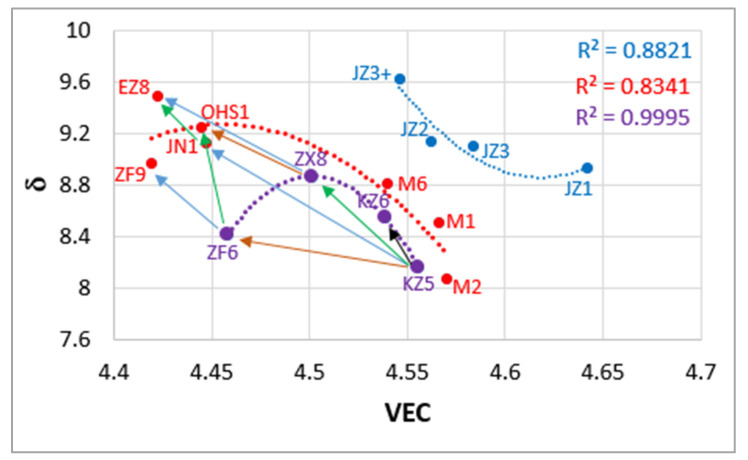
δ versus VEC map of Nb-silicide-based alloys with addition of Al, Cr, Ge, Hf, Sn, Ta, W. Arrows indicate addition of Hf (blue), Ta (black), Sn (green), Ge (brown). R^2^ values for parabolic fit of data. Purple and red data for Ti rich alloys with KZ5 as the “basis” alloy, blue data for JZ series alloys. Nominal compositions (at.%) KZ5 = Nb-24Ti-18Si-5Al-5Cr, KZ6 = KZ5+6Ta, ZX8 = KZ5+5Sn, ZF6 = KZ5+5Ge, JN1 = KZ5+5Hf, ZF9 = KZ6+5Hf, EZ8 = JN1+5Sn, OHS1 = KZ5+5Ge+5Sn [10,13,35,36,38,47], see Appendix A. Actual compositions (at.%) M1 = Nb-24.8Ti-17.6Si-0.2Ge-5.3Cr-2Hf-1.9Al-0.3Sn, M2 = Nb-26Ti-12.6Si-4.9Ge-6.7Cr-1.9Hf-1.9Al-0.5Sn, M3 = Nb-24.76Ti-16.8Si-4.2Ge-6.2Cr-1.4Hf-1.9Al-1.5Sn [9,31,46].

Figure 15 is the δ versus VEC map of Nb-silicide-based alloys with addition of Al, Cr, Ge, Hf, Sn, Ta, W. With the exception of the alloys JZ1, JZ2, JZ3 and JZ3+, all other alloys are Ti rich (Ti ≥ 24 at.%). Notice differences in the concentrations of Al, Cr, Ge, Hf and Sn in the alloys M1, M2 and M6 [9,31,46] compared with the other Ti rich alloys. The data show (i) a clear separation between Ti lean and Ti rich Nb-silicide-based alloys that are B free (this separation is different from that discussed in [19], where Ti-rich Nb-silicide-based alloys with B addition form their own separate group), (ii) that improvement in oxidation resistance (see [5] and the data in this work for JZ1 to JZ3+) goes together with the decrease and increase, respectively of the parameters VEC and δ, in agreement with NICE [4], (iii) that the addition of specific elements, e.g., Ge, Hf, Sn, Ta brings changes of the aforementioned parameters along “different routes” indicated by the arrows in Figure 15, with Hf having a remarkable effect, particularly in synergy with Sn (iv) and that this map “pinpoints” an area that could be exploited by alloy design/development to meet property goals (see Section 6).

### 5.4. Oxidation

#### 5.4.1. Oxidation at 800 °C

The weight change of the alloy JZ3 was 14.92 mg/cm^2^. The alloy followed linear oxidation (Table 3), similar to the alloys JZ1 and JZ2 [14]. Compared with the alloy JZ2, the addition of Al and Cr in the alloy JZ3 (a) did not change the oxidation kinetics, (b) reduced the linear oxidation rate by an order of magnitude and (c) eliminated pest oxidation. It should be noted that the alloy JZ2, which partially pested after 100 h, had five times and twenty times more vol.% Nb_ss_ than the alloys JZ3 and JZ3+, respectively.

The oxidation of the alloy JZ3+ accelerated after 40 h and resulted to pest oxidation after 74 h (Figure 8b). Considering the actual compositions of both alloys, the pesting of JZ3+ would suggest that in Ta and W containing alloys there is an upper limit to the concentration of Sn above which the benefit of Sn on oxidation is lost. The alloy JZ3+ had a lower vol.% Nb_ss_ and a higher vol.% of intermetallics, in particular Nb_5_Si_3_, compared with JZ3. After the preparation of specimens for the oxidation experiments it was noted that there were microcracks in the specimens of JZ3+ but not in the specimens of the alloy JZ3. The formation of microcracks in JZ3+ was attributed to the high hardness of the alloyed Nb_5_Si_3_ (see previous section) and W-rich solid solution, and the high vol.% of the silicide. The presence of cracks is often considered a reason for the pest oxidation of intermetallics [48]. In other words, the upper limit of Sn content in the alloy is imposed by the alloying elements and their concentrations in Nb_5_Si_3_ and Nb_ss_ and the vol.% of both phases. We shall revisit these points at the end of this section, after we consider first the oxidation of the non-pesting alloy JZ3.

Current knowledge about how the synergy of Al, Cr, Ge, Hf, Sn or Ti with/without RM affects the oxidation of Nb-silicide-based alloys in the pest regime is based on experimental work for Ti-rich alloys with Ti ≥ 24 at.% [4,5,11,12,13,24,25,49,50,51] for which the literature would suggest (1) that the addition of Ge or Sn with Al and Cr is effective at suppressing pest oxidation at 800 °C, (2) that the addition of Ge with Al and Cr is more effective than that of Sn with the Al and Cr at 800 °C, (3) that the simultaneous addition of Ge and Sn with Al and Cr is also beneficial to oxidation in the pest regime, (4) that thinner scales are formed on alloys when Al and Cr are present simultaneously [49] with/without the simultaneous presence of Ge and Sn [13] compared with alloys with Al or Cr addition [49], (5) that the addition of 6 at.%Ta is detrimental to oxidation resistance at 800 °C and promotes linear kinetics [49], (6) that the improved oxidation of Sn containing alloys at 800 °C and the suppression of pest oxidation is linked (a) with the lower vol% of Nb_ss_ and (b) the stability of A15-Nb_3_X in the alloy [12,13,24,25,50], (7) that there is a “critical” Sn concentration below which the A15-Nb_3_Sn is not stable [11,24,51], (8) that in Ge containing non-pesting alloys no A15 compound was present in their microstructures [11], (9) that improved oxidation behaviour at 800 °C is linked with low vol% of Nb_ss_ [11,35,37,52], (10) that suppression of pest oxidation is linked with enrichment of the substrate just below the scale/substrate interface with Sn and Ge and the formation of A15 compound(s), (11) that the C14-Cr_2_Nb Laves phase probably does not benefit oxidation at 800 °C in alloys with simultaneous addition of Sn with Al and Cr [13], (12) that Cr and Al with/without Ta impede the diffusion of oxygen toward the bulk of the alloys [49] and (13) that the diffusion zone that forms in alloys with low Sn content is significantly thicker than that formed in alloys with high Sn [10,24].

Both alloys “complied” with most of the points (1) to (13), nonetheless the alloy JZ3 did not pest whereas JZ3+ did. The Nb_ss_, Nb_5_Si_3_, C14-Cr_2_Nb and A15 phases were present in the microstructure of the oxidation specimen of the alloy JZ3 at the start of the isothermal oxidation together with Ti and Cr-rich A15 and Ti-rich Nb_5_Si_3_. After the isothermal oxidation at 800 °C, compared with JZ3-AC, in the bulk of the oxidised alloy (a) the Cr-rich A15 was not observed, (b) the Nb_ss_ was poorer in W (Ta/W ≈ 1), (c) the Laves was poorer in Cr, (d) the Nb_5_Si_3_ and Ti-rich Nb_5_Si_3_ essentially had the same composition and (e) the Ti-rich A15 was poorer in Ti and Cr (Table 7). There was no diffusion zone in JZ3, and the scale was cracked along the edges of the specimen (Figure 8a) and was thinner compared with that on the alloy KZ6 (Nb-24Ti-18Si-5Al-5Cr-6Ta [36,49]). There was no enrichment in Ge and/or Sn in the substrate just below the substrate/scale interface (the A15 and Ti-rich A15 were already present in the microstructure).

Considering the oxidation of the Ti rich and Ta containing alloys KZ6 (Nb-24Ti-18Si-6Ta-5Al-5Cr) and KZ8 (Nb-24Ti-18Si-6Ta-8Cr-4Al) [36,49] and the Al, Cr, Ge and Sn containing alloy OHS1 (Nb-24Ti-18Si-5Al-5Cr-5Ge-5Sn) [13], the linear oxidation kinetics of the alloy JZ3 were attributed to the concentration of Ta (point 5) and the synergy of Ta with W. The suppression of pest oxidation in the alloy JZ3 was attributed to it meeting the points (1) to (4), (6) to (9) and (12).

The same phases were present in the microstructure of the alloys JZ3+ and JZ3. However, there were some important differences in the compositions of the phases. The solid solution in the alloy JZ3+ was significantly richer in W with W/Ta = 2.3 and this ratio increased slightly after exposure to high temperature. As a consequence of its high refractory metal content the Ti concentration in the solid solution in JZ3+ was significantly lower than that in the solid solution in the alloy JZ3. The lower Ti concentration in the Nb_ss_ in the alloy JZ3+ resulted to lower Al and Cr concentrations in the solid solution compared with the alloy JZ3 (in Nb-silicide-based alloys the solubility of Al or Cr in Nb_ss_ increases with increasing concentration of Ti in the solid solution [4,38]). The same was also the case for the Sn and Ge concentrations in the solid solution in JZ3+ that also decreased after exposure to high temperature. Thus, even though the alloy JZ3 had almost four times the vol.% of Nb_ss_ compared with the alloy JZ3+, the solid solution in the cast microstructure of the latter was poorer regarding the elements that are known to improve the oxidation resistance of the solid solution [21] and to decrease the diffusivity of oxygen in it [4], and became even poorer after exposure to high temperature, according to the data for [JZ3+]-HT (see Tables S3 and S4).

The A15 compound in the alloy JZ3 formed as normal A15, Ti-rich A15 and Cr-rich A15 (Table S1). The former two had lower W/Ta ratio, lower Ti, Cr and Sn and higher Ge content compared with the alloy JZ3+ in which the Cr-rich A15 did not form. The Cr-rich A15 was rich in Ti, Al, Ge and Hf. After exposure to high temperature the compositions of the A15 in both alloys were essentially the same with the exception of the concentrations of Sn and Ge that respectively increased and decreased in [JZ3+]-HT. Furthermore, after exposure to high temperature the vol.% of the A15 increased in the alloy JZ3 but did not change in the alloy JZ3+ (Table 1).

The vol.% of the Nb_ss_ and Nb_5_Si_3_ in the alloy JZ3+ respectively was lower and higher than that in the alloy JZ3. In the early stages of oxidation at 800 °C the weight change of JZ3+ was lower than that in JZ3 (Figure 7). The better oxidation behaviour of the alloy JZ3+ for the first 40 h of exposure to 800 °C compared with the alloy JZ3 was attributed to the volume fractions of its phases. After this time the detrimental effect of micro-cracking dominated, and the alloy was not able to maintain the low weight change. Instead, the weight gain increased dramatically and the specimen disintegrated into powder.

Compared with the alloys JZ2 [14], OHS1 [13] and ZX8 [10], whose scales consisted of Nb-rich, Si-rich and Ti-rich oxides, the latter oxide type was not observed in the scale of JZ3. In this alloy, both the Nb-rich and Si-rich oxides were lean in Ge, Sn, Ta and Ti and the Si-rich oxide was also lean in Cr and W.

#### 5.4.2. Oxidation at 1200 °C

The alloy JZ3 followed parabolic kinetics at the early stages of the isothermal oxidation (≤9 h) and linear kinetics thereafter, as was the case for the alloys JZ1, JZ2 [14]. Both the rate constants of JZ3 were one order of magnitude lower than those of JZ2. The alloy JZ3+ followed only parabolic kinetics and its parabolic rate constants (Table 3) were about two orders of magnitude higher than that of the single crystal Ni-based superalloy CMSX-4, which is about 4 × 10^−12^ g^2^ cm^−4^ s^−1^ after 100 h isothermal oxidation at 1200 °C (weight change about 4 mg/cm^2^), and one order of magnitude higher for the first 14 h of oxidation. The weight change of JZ3+ was four times that of CMSX-4. The alloy JZ3+ matched the oxidation of the “best” Nb-silicide-based alloys [5] but remarkably with less Ti content and with the addition of two RM.

Compared with the alloy JZ2 [14], the oxide scales formed on the alloys JZ3 and JZ3+ were thinner, in particular the scale that formed on JZ3+. The oxidation specimen of JZ3+ contained micro-cracks, yet the oxidation of this alloy was better than that of JZ3. The oxide scales that formed on JZ3 and JZ3+ consisted of the same oxides, the chemical compositions of which were similar. However, it should be noted (i) that there were differences in the compositions of the phases (a) in the starting (i.e., as cast) microstructures, and (b) in the diffusion zones (see below and Table 7), (ii) that after the heat treatment at 1500 °C there was contamination by oxygen of the substrate below the scale only in the alloy JZ3 and (iii) that the contamination by oxygen of the phases in the diffusion zone of JZ3+ was less severe compared with the diffusion zone in JZ3. Both (ii) and (iii) are consistent with lower diffusivity of oxygen in JZ3+ (which also had lower vol.% Nb_ss_ and A15 and thus higher vol.% Nb_5_Si_3_ than JZ3 (Table 1)).

The scale that formed on JZ3+ was thin and well adherent. Protective oxide scales grow slowly with high integrity on the substrate surface. The rate determining step is the diffusion of cations and/or anions of the oxide forming elements and of electrons via the lattice defects in the scale. Slow oxide growth results to low k_p_ values. The parabolic rate constant of JZ3+ was similar to that of the best oxidation resistant Ti rich (≥24 at.%) Nb-silicide-based alloys [5].

Similarly with the oxidation in the pest regime, current knowledge about how the synergy of Al, Cr, Ge, Hf, Sn or Ti with/without RM affects the oxidation of Nb-silicide-based alloys at high temperatures is from research based on Ti-rich alloys with Ti ≥ 24 at.% and Hf-rich (≤8 at.%) alloys [4,5,10,11,12,13,24,25,49,50,51,53]. Tin addition (2–8 at.%) to MASC type alloys (MASC = Nb-25Ti-16Si-8Hf-2Al-2Cr [3,16,53]) improved their oxidation resistance at 1200 °C compared with Sn-free MASC and all alloys gained weight more than 50 mg/cm^2^ after 50 h exposure [12]. The mass gain of the alloy Nb-24Ti-18Si-5Ge-5Al-5Cr was about 40 mg/cm^2^ after 100 h [11]. Addition of 5 at.% Sn to Nb-24Ti-18Si-2Mo-5Al-5Cr-5Hf-5Sn reduced the oxidation rate at 1200 °C but the alloy still gained weight of about 90 mg/cm^2^ after 100 h [25]. Comparison of the data for the alloys mentioned above with that for the alloys JZ2 [14], JZ3 and JZ3+ shows (1) that the adherence of the oxide scale at 1200 °C improved when Al and Cr were in synergy with Ta, W, Ge and Sn with low Hf (≤1 at.%) and Ti (≤12 at.%) concentration, (2) that oxidation resistance improved with the increase in the content of Sn in the alloy JZ3+, (3) that even though individual addition of Sn or Ge improves oxidation resistance at 1200 °C but not the spallation of the scale [10,11], their simultaneous presence in the alloys JZ3 and JZ3+ improved their oxidation behaviour, particularly in the latter alloy and (4) that the suppression of scale spallation that was observed in the alloy OHS1 [13] was not destroyed by the synergy of Al, Cr, Ge and Sn with Hf, Ta and W. The latter synergy also improved the oxidation kinetics in JZ3+ compared with the Ta and W free alloy OHS1 (parabolic + linear in OHS1, with the k_p_ for the first 3h of oxidation [13] similar to that of JZ3+ for the whole duration of the oxidation experiment).

In Ti-rich (Ti ≥ 24 at.%) Sn-containing Nb-silicide-based alloys, after isothermal oxidation at 1200 °C there was enrichment in Sn in the substrate at the oxide scale/substrate interface where the compounds Nb_3_Sn and Nb_5_Sn_2_Si were formed [10,12,24,25]. The latter intermetallic has W_5_Si_3_ as its prototype (the same as βNb_5_Si_3_), and is not believed to be stable in the Nb-Si-Sn ternary system at 1200 °C [54]. The research in [10,12,24,25] would suggest that the synergy of Mo and Hf with Al and Cr, and contamination of the alloy by oxygen stabilised the Nb_5_Sn_2_Si compound at high temperatures. The Nb_5_Sn_2_Si or other TM_5_Sn_2_X compounds [55] (TM = Nb, Ti, X = Al, Si) were not observed in the alloys JZ2, JZ3 and JZ3+ at 1200 °C (Table 7), which would suggest that the synergy of Sn with Ge, Ta and W did not stabilise these compounds. Segregation of solutes at the substrate just below the scale/substrate interface has been studied in [10,13,24,25,50] and reported in [12]. Theories that can account for the segregation of Al, Cr, Ge, Hf, Si, Sn and Ti below the scale/substrate interface were discussed in [24].

Common observations in the alloys JZ2 [14], JZ3 and JZ3+ were (i) that the oxide scales were free of Sn and Ge and (ii) that Sn and Ge containing intermetallic phases formed in the diffusion zone (Table 7). The same phases formed in the diffusion zones of JZ3 and JZ3+ but there were differences with the phases in the diffusion zone of JZ2 [14] and OHS1 (Table 7). Furthermore, the Sn or Ge rich layers that were formed in the alloy JZ2 were not observed in JZ3 and JZ3+. There was a gradual increase and decrease, respectively of the W/Ta and RM/(Ti+Hf) ratios of the solid solution and Nb_5_Si_3_ from the alloy JZ2 to JZ3+, and a decrease of the RM/(Ti+Hf) ratio of Nb_5_(Si,Sn)_3_ from the alloy JZ3 to JZ3+, with essentially no significant changes of the <Si> = Al+Ge+Si+Sn content of the silicides. Compared with the alloy JZ2, which formed two diffusion zones, in the alloys JZ3 and JZ3+ only one diffusion zone formed. In the alloy JZ2 the Nb_5_(Si_1-x_,Ge_x_)_3_ and NbGe_2_ compounds and Sn-rich and Ge-rich layers formed in the diffusion zone 1 (zone immediately below the scale) and Nb_5_Si_3_, Ti-rich Nb_5_Si_3_, A15 and Nb_ss_ in diffusion zone 2, whereas in the alloys JZ3 and JZ3+ the Nb_5_Si_3_, Nb_5_(Si,Sn)_3_, Nb_5_(Si_1-x_,Ge_x_)_3_, A15 and (Nb,W)_ss_ were formed. The solid solution in the diffusion zones of JZ3 and JZ3+ was very rich in W (W/Ta = 3.15 and 3.64, respectively in JZ3 and JZ3+, Table 7) and essentially Ti free (Table S7). It should be noted that only the starting microstructure of JZ3+ had a very W-rich Nb_ss_ (W/Ta = 2.3) compared with the Nb_ss_ in JZ3 (W/Ta = 1). During oxidation the solid solution switched from being Nb-rich (Nb/W = 3.7 and 0.9, respectively in JZ3 and JZ3+ (19.8 in JZ2)) to W-rich (Nb/W = 0.27 and 0.24, respectively in JZ3 and JZ3+ (9.8 in JZ2)), in other words, the increase of Sn concentration and the addition of Al and Cr resulted to significant changes in the chemical composition of the solid solution in the alloys JZ3 and JZ3+, compared with JZ2.

In Sn-containing alloys the oxide scales spall off after oxidation [10,12,24,25,50] and the same is the case for Ge-containing alloys [11]. The alloying of Nb_5_Si_3_ with Ti or Sn reduces its hardness, in contrast with Ge that has the opposite effect [32]. Compared with JZ3, the Nb_5_Si_3_ and Nb_5_(Si,Sn)_3_ in the diffusion zone of JZ3+ had lower RM/(Ti+Hf) ratios and the latter silicide also was richer in Ti and Sn (Tables S6 and S7 and Table 7) whereas in the Nb_5_(Si,Ge)_3_ the RM/(Ti+Hf) ratio did not differ significantly in the two alloys (Table 7). Furthermore, in the alloy JZ2 the Nb_5_(Si_1-x_,Ge_x_)_3_, NbGe_2_, Sn-rich areas and Ge-rich areas formed below the scale, not the Nb_5_(Si,Sn)_3_ (see above). The improved adhesion of the oxide scale on JZ3+ was attributed (i) to the thickness of the scale and (ii) the improved deformability of the substrate below the thin scale.

The encouraging result of the research presented in this paper is that a Ti lean Nb-silicide-based alloy with simultaneous addition of RM (essential for strength and creep [4,5]), Al, Cr, Ge, Hf and Sn (essential for oxidation resistance [4,5]) can “match” the high temperature oxidation of the best oxidation resistant Ti rich alloys, some of which are B-containing alloys [5]. Undeniably, the inferior oxidation performance in the pest regime is a matter of concern, and must be addressed by future alloy development research.

#### 5.4.3. Comparison with RCCAs

The oxidation of Nb-silicide-based alloys was compared with that of RCCAs in [5]. The isothermal oxidation of RCCAs has been reported for periods that vary from 1 h to 100 h [2]. The majority of the data for RCCAs are for 1000 or 1100 °C. Less data are available for 1300 °C and the data are for up to 50 h. There is one study at 800 °C. Data for the oxidation of RCCAs in the pest regime is rare and papers do not indicate whether the RCCAs experienced pest oxidation, albeit with some exceptions, for example the alloy AlNbTiVZr_0.25_ disintegrated after 50 h at 900 °C [56]. Only the oxidation of one RCCA is directly comparable with the alloys JZ3 and JZ3+. The mass change of Al_9.2_Cr_5.7_Hf_0.5_Mo_1.3_Nb_47_Ti_25.9_V_9.6_W_0.8_ (note that the Cr, Mo and Ti concentrations of this RCCA are similar to those in many Nb-silicide-based alloys, and that the Al content is almost double) was 2, 28 mg/cm^2^ and 32 mg/cm^2^, respectively at 800 °C, 1100 °C and 1200 °C, after 5 h at each temperature [2] but it was not reported whether this alloy suffered from pest oxidation at 800 °C. Compared with the data in Table 3, the oxidation of the aforementioned RCCA is significantly inferior. Furthermore, the oxidation of both alloys is superior to that of the RCCAs studied to date [2]. It has not been reported whether this alloy suffered from pest oxidation at 800 °C. Compared with the data in Table 3, the oxidation of the aforementioned RCCA is significantly inferior. Furthermore, compared with the data reviewed in [2], the oxidation of both alloys is superior to that of RCCAs studied to date.

## 6. Comparisons of Experimental Data with NICE

### 6.1. Macrosegregation of Si

We used the actual compositions of the alloys that were given in the Table 2, and NICE to calculate MACSi in the alloys JZ3 and JZ3+. The calculated MACSi was 4.6 and 3.5 at.%, compared with the experimental values of 4 and 3.1 at.%, respectively. Even though NICE slightly over-estimated MACSi for both alloys, the agreement with the experimental results is considered to be good. Furthermore, NICE predicted the correct trend of MACSi with increase of Sn concentration in alloys where Sn and Ge were in synergy with RM and TM additions and with Al and Cr (MACSi_experimental_ of 5.6, 4.9, 4 and 3.1 at.%, compared with MACSi_calculated_ 4.8, 5.2, 4.6, 3.5 at.%, respectively for the alloys JZ1, JZ2, JZ3 and JZ3+, Table 8). To our knowledge, this is the first time that research has shown (i) that Nb-silicide-based alloys with simultaneous additions of RM, TM, Al, Cr, Ge and Sn have low Si macrosegregation and (ii) that the decreasing trend in MACSi with such alloying additions can be predicted correctly by an alloy design methodology.

### 6.2. Volume Fraction of Solid Solution

The calculated vol.% of Nb_ss_ (using NICE and the actual compositions of the cast alloys in the Table 2) was 9.9 and 7.4%, respectively in JZ3 and JZ3+, higher than the experimental values, both of which, it should be noted, were for the heat treated alloys (Table 1). NICE predicted the correct trend for the fraction of solid solution in alloys where Sn and Ge were in synergy with RM and TM additions and with/without Al and Cr (vol.%Nb_ss_^experimental^ 46.5, 34.5, 4.7, 1.2 compared with vol.%Nb_ss_^calculated^ 44.1, 34.8, 9.9, 7.4, respectively for the alloys JZ1, JZ2, JZ3 and JZ3+, Table 8). In general, the vol.% of Nb_ss_ in alloys with RM, TM and simple metal and metalloid element (Al, Ge, Si, Sn) addition tends to decrease after heat treatment. Thus, most likely the difference between the experimental and calculated values of the vol.% Nb_ss_ in the cast alloys JZ3 and JZ3+ would not be as high if experimental data for the cast alloys were available.

### 6.3. Chemical Composition of Solid Solution

The chemical composition of the solid solution in the cast alloys also was calculated using the actual alloy compositions given in the Table 2 and the relationships in NICE that link the concentration of a solute element in the alloy and its solid solution [4]. The calculated compositions were 40.7Nb-15Ti-3Si-10.5Ta-6.2W-0.7Hf-4.4Sn-1.4Ge-6.6Al-11.5Cr and 28.6Nb-10.8Ti-3.8Si-10Ta-28.3W-0.14Hf-1.5Sn-1.4Ge-4Al-11.5Cr, respectively for the alloys JZ3 and JZ3+. Similarly to the alloys JZ1 and JZ2 [14], the Ta concentration was calculated using data for Mo in NICE, because there is not enough experimental data available for Ta containing Nb-silicide based alloys. The calculated composition of the solid solution in JZ3 should be compared with Nb_ss_^c^ in the Table S1. The calculated compositions of both the solid solutions are considered to be in good agreement with the experimental data (Tables S1 and S3).

#### The Parameters VEC, δ and Δχ of the Solid Solution

The parameters VEC, δ and Δχ of the calculated solid solutions respectively were 4.8, 5.45 and 0.2125, and 5.1436, 8.514 and 0.348 for the alloys JZ3 and JZ3+, and were consistent with [26]. Furthermore, both the δ values were higher than 5, which is typical for a solid solution that is not Si-free [4,5,26]. Note that Figure 5b in [26] shows (a) that the demarcation between Si-free Nb_ss_ and Ti-rich Nb_ss_ is δ ≈ 5, (b) that Si-free Nb_ss_ has δ less than about 5 and (c) that the Figure 5 in [26] does not include alloys with simultaneous addition of Ta, W, Ge and Sn, with/out Al and Cr. The values of the aforementioned parameters for the experimental compositions of the solid solutions in the two alloys were also consistent with NICE. Indeed, for the Nb_ss_^c^ in the alloy JZ3 the parameters VEC, δ and Δχ were 4.916, 6.03 and 0.2524, respectively, and for the (Nb,W)_ss_ in JZ3+ they were 5.288, 4.87 and 0.3727, respectively. Note that the value of the parameter δ in the (Nb,W)_ss_ in JZ3+ is very close to δ ≈ 5, which would suggest that Si-free solid solution could form in the alloy JZ3+. This was confirmed after the heat treatment (see Section 4.1), where Si-free (Nb,W)_ss_ was formed in [JZ3+]-HT with values of the parameters VEC, δ and Δχ 5.377, 4.239 and 0.3687, respectively, and with δ reduced significantly below δ ≈ 5. The values of the parameters VEC and δ of the experimental and calculated solid solutions in the alloys JZ3 and JZ3+ were in agreement with the ranges in NICE [4,26]. The Δχ values for the experimental solid solutions showed that the range of the parameter Δχ of the solid solution is wider than that given in [26] when the data for alloys with the simultaneous addition of Ta, W, Ge and Sn, with/without Al and Cr, is included. This conclusion was also supported by the calculated compositions of the solid solutions. It should also be noted that the data for the solid solution in JZ3+ confirmed for the first time that Si-free Nb_ss_ also can form in Ti poor Nb-silicide-based alloys with simultaneous additions of Al, Cr, Ge, Hf, Ta and W.

### 6.4. Composition of Nb_5_Si_3_

In the Nb_5_Si_3_ database in NICE the data for RM additions in tetragonal Nb_5_Si_3_ is limited. This lack of data hampered the calculation of the concentrations of Ta and W in the silicide. Calculation of the concentrations of the other elements in the alloyed Nb_5_Si_3_ was possible. For example, using the actual composition of the alloy JZ3+, the calculated compositions of the normal and Ti-rich Nb_5_Si_3_ were 38.5(Nb+Ta+W)-19Ti-31.2Si-1.4Sn-6.1Ge-1Hf-2.6Al-0.2Cr and 30.2 (Nb+Ta+W)-25.7Ti-17.4Si-9.9Sn-5.8Ge-2.2Hf-4.1Al-4.7Cr, respectively. Compared with the data in Table S3, the Ti and Cr concentrations in the normal Nb_5_Si_3_ were overestimated and underestimated, respectively whereas in the Ti-rich Nb_5_Si_3_ the Ti and Al concentrations were underestimated and overestimated, respectively.

### 6.5. Weight Change in Isothermal Oxidation

The average weight changes of the alloys JZ3 and JZ3+ at 800 °C and 1200 °C that were calculated using NICE, were 9.9 and 7.4 mg/cm^2^, and 42 and 15 mg/cm^2^, respectively at each temperature (Table 8). Compared with the data in Table 3, NICE underestimated the ΔW/A values for both alloys at 800 °C and overestimated at 1200 °C. For the latter temperature, the agreement between calculated and experimental weight change for JZ3+ is exceptional. The better oxidation behaviour of JZ3+ compared with JZ3 at 1200 °C correlated well with the decrease in VEC and increase in δ parameter values (see next section), as required by NICE [4,10,11,13,24]. Furthermore, NICE predicted the correct trend of oxidation resistance with increase of Sn concentration in alloys where Sn and Ge were in synergy with RM and TM additions and with Al and Cr (Table 8).

NICE cannot predict whether an alloy would suffer from catastrophic pest oxidation. For a designed alloy, when the calculated vol.% of the Nb_ss_ is low (<10%) it is anticipated that oxidation will improve both in the pest regime and at high temperatures but at the same time attention is drawn to the fact that the fracture toughness of the alloy, which NICE cannot calculate, will be lower compared with alloys with higher vol.% Nb_ss_.

### 6.6. Calculated Creep Rate for Creep Goal

The values of the parameters VEC, Δχ, δ and the ratios sd/sp (sd electronic configuration elements over sp electronic configuration elements) and Nb/(Ti+Hf) that were calculated for the actual compositions of the cast alloys JZ3 and JZ3+, respectively were 4.584, 0.1942, 9.1, 2.24, 3.11 and 4.546, 0.1932, 9.63, 1.89 and 2.95 (Table 9). Both alloys met the constraint (a) (Section 2.2.1). The calculated creep rates at 1200 °C and 170 MPa using NICE and the values of the above parameters and ratios were in the ranges 5.8 × 10^−6^ s^−1^ to 4.3 × 10^−8^ s^−1^, and 1.2 × 10^−5^ s^−1^ to 5.1 × 10^−8^ s^−1^, with average creep rates 9.8 × 10^−7^ s^−1^ and 1.95 × 10^−6^ s^−1^, respectively for the alloys JZ3 and JZ3+. The average rates were lower than that of the single crystal Ni-based superalloy CMSX-4 for the same conditions (5.6 × 10^−5^ s^−1^) and higher than the creep rate of 1 × 10^−7^ s^−1^ that is the criterion in NICE to predict whether it is likely for a designed (selected) alloy to meet the creep goal [4]. However, it should be noted that the calculated creep rates of 4.3 × 10^−8^ s^−1^ and 5.1 × 10^−8^ s^−1^, respectively for JZ3 and JZ3+ were lower than 1 × 10^−7^ s^−1^. The average calculated creep rates of the alloys JZ3 and JZ3+ were higher than those of the alloys JZ1 and JZ2 [14], which is expected given the effect that Al and Cr additions have on the creep of Nb-silicide-based alloys [4,5], the trends in the parameters VEC, Δχ, δ, sd/sp and Nb/(Ti+Hf) for oxidation resistance and creep resistance [4].

## 7. Summary and Concluding Remarks

We studied the microstructures and properties of the alloys JZ3 and JZ3+. The densities of both alloys were lower than those of the Ni-based superalloys developed to date. Both alloys had Si macrosegregation and the same phases in their as cast and heat treated microstructures, namely βNb_5_Si_3_, αNb_5_Si_3_, A15-Nb_3_X (X = Al,Ge,Si,Sn), C14-Cr_2_Nb and solid solution. W-rich solid solutions were stable in both alloys. At 800 °C only the alloy JZ3 did not suffer from catastrophic pest oxidation, and at 1200 °C a thin and well adhering scale formed only on JZ3+. The latter alloy followed parabolic oxidation with rate constant one order of magnitude higher than the single crystal Ni-superalloy CMSX-4 for the first 14 h of oxidation. The oxidation of both alloys was superior to that of RCCAs. Both alloys were predicted to have better creep at the creep goal condition compared with the superalloy CMSX-4.

In our opinion, the notable outcomes of this study are the following ones. (a) Nb-silicide-based alloys with simultaneous addition of RM (Ta,W), TM (Cr,Hf,Ti), Al, Ge and Sn to achieve a balance of properties with low density can have low Si macrosegregation. (b) A Ti lean Nb-silicide-based alloy with simultaneous addition of Ta and W (essential for strength and creep), and Al, Cr, Ge, Hf and Sn (essential for oxidation resistance) can match the high temperature oxidation of the best oxidation resistant Ti rich alloys. (c) Data about Si macrosegregation, solid solution volume fractions, chemical composition of solid solution and Nb_5_Si_3_, weight changes in isothermal oxidation at 800 and 1200 °C can be calculated with confidence using the alloy design methodology NICE.

## Figures and Tables

**Table 1:** Density of the as cast (AC) and % area of Nb_ss_ and A15-Nb_3_X in the bulk of the AC and heat treated (HT) alloys JZ3 and JZ3+.

Alloy	Density (g/cm^3^)	Nb_ss_ (%)	A15-Nb_3_X (%)
JZ3-AC	7.94 ± 0.01 7.93 − 7.96	-	26.8 ± 2.3 24.2 − 28.8
JZ3-HT ^a^	-	4.7 ± 0.7 4.1 − 5.5	31.2 ± 3.4 27.5 − 34.2
[JZ3+]-AC	7.54 ± 0.01 7.53 − 7.55	-	13.3 ± 0.3 12.9 − 13.5
[JZ3+]-HT ^a^	-	1.2 ± 0.2 1.1 − 1.4	13.9 ± 0.9 12.9 − 14.6

^a^ data includes a small percentage of hafnia owing to its same contrast with the solid solution.

**Table 2:** Average chemical compositions (at.%) and summary of phases in as cast and heat treated alloys JZ3 and JZ3+.

Alloy	
JZ3	JZ3+
As Cast	
Nb-12.4Ti-17.7Si-6Ta-2.7W-3.7Sn-4.8Ge-1Hf-4.7Al-5.2Cr	Nb-12.4Ti-19.7Si-5.7Ta-2.3W-5.7Sn-4.9Ge-0.8Hf-4.6Al-5.2Cr
Nb_ss_	(Nb,W)_ss_
Nb_5_Si_3_, Ti-rich Nb_5_Si_3_	Nb_5_Si_3_, Ti-rich Nb_5_Si_3_
A15-Nb_3_X, Ti-rich A15, Cr-rich A15	A15-Nb_3_X, Ti-rich A15
C14-NbCr_2_ Laves	C14-NbCr_2_ Laves
HfO_2_	HfO_2_
Heat Treated	
Nb-12.8Ti-18.3Si-5.7Ta-2.5W-3.4Sn-5.2Ge-0.8Hf-4.8Al-4.8Cr	Nb-12.3Ti-20.7Si-5.7Ta-2W-4.8Sn-5.1Ge-0.8Hf-4.6Al-4.7Cr
Nb_ss_	(Nb,W)_ss_
Nb_5_Si_3_, Ti-rich Nb_5_Si_3_	Nb_5_Si_3_, Ti-rich Nb_5_Si_3_
A15-Nb_3_X	A15-Nb_3_X
C14-NbCr_2_ Laves	C14-NbCr_2_ Laves
HfO_2_	HfO_2_

**Table 3:** Weight gains and linear (k_l_) and parabolic (k_p_) oxidation rate constants of the alloys JZ3 and JZ3+ after isothermal oxidation at 800 and 1200 °C for 100 h.

Alloy	800 °C		1200 °C		
	Weight Gain (mg/cm^2^)	Rate Constant	Weight Gain (mg/cm^2^)	Rate Constant	
		kl (g cm^−2^ s^−1^)		kl (g cm^−2^ s^−1^)	kp (g^2^ cm^−4^ s^−1^)
JZ3	14.9 (100 h)	4.4 × 10^−8^ (0–100 h)	24.2 (100 h)	6 × 10^−8^ (9–100 h)	5.9 × 10^−10^ (0–9 h)
JZ3+	13.9 (74 h)	6.5 × 10^−9^ (0–40 h) 1.2 × 10^−7^ (40–74 h)	14 (100 h)		5.5 × 10^−10^ (0–100 h) 4.7 × 10^−11^ (0–14 h) 5.8 × 10^−10^ (6–100 h)

**Table 4:** Alloy parameters for the macrosegregation of Si in the cast alloys KZ5, KZ6, JZ3, OHS1.

Alloy	ΔH_m_ (kJ/mol)	T_m_ (K)	ΔH_m_/T_m_(J/molK)		ΔH_m_^sd^/ΔH_m_^sp^		T_m_^sd^ (K)		T_m_^sp^ (K)		T_m_^sd^/T_m_^sp^		[ΔH_m_/T_m_] × [ΔH_m_^sd^/ΔH_m_^sp^]^−1^		MACSi (at.%)	
OHS1	27.7	2090	13.25	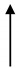	1.37	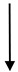	1653	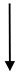	437	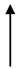	3.8	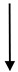	9.67	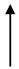	6.8	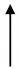
JZ3	29.1	2242	12.98		1.6		1823		419		4.35		8.11		4	
KZ6	28.6	2257	12.67		1.86		1894		363		5.23		6.81		2.5	
KZ5	27.5	2239	12.28		2.05		1909		330		5.78		5.99		1.3	

KZ5 = Nb-18Si-24Ti-5Al-5Cr, KZ6 = Nb-18Si-24Ti-6Ta-5Al-5Cr [36], OHS1 = Nb-18Si-24Ti-5Al-5Cr-5Ge-5Sn [13] (nominal compositions, Appendix A).

**Table 5:** Alloy parameters for the macrosegregation of Si in the cast alloys JZ3, ZF5 and KZ7.

Alloy	ΔH_m_ (kJ/mol)	T_m_ (K)	ΔH_m_/T_m_ (J/molK)		ΔH_m_^sd^/ΔH_m_^sp^		T_m_^sd^ (K)	T_m_^sp^ (K)		T_m_^sd^/T_m_^sp^		[ΔH_m_/T_m_] × [ΔH_m_^sd^/ΔH_m_^sp^]^−1^		MACSi (at.%)	
JZ3	29.1	2242	12.98	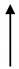	**1.6**	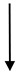	1823	419	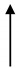	4.35	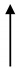	8.11	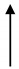	4	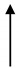
ZF5 (Al)	28	2202	12.72		1.72		1820	382		4.76		7.39		2.9	
KZ7 (Al)	27.7	2272	12.19		2.15		1948	324		5.78		6		2.3	

KZ7 = Nb-24Ti-18Si-5Al [36], ZF5 = Nb-24Ti-18Si-5Al-5Ge [35] (nominal compositions).

**Table 6:** Alloy parameters for the macrosegregation of Si in the cast alloys KZ4, JZ3 and OHS1.

Alloy	ΔH_m_ (kJ/mol)	T_m_ (K)		ΔH_m_/T_m_ (J/molK)		ΔH_m_^sd^/ΔH_m_^sp^		T_m_^sd^ (K)		T_m_^sp^ (K)		T_m_^sd^/T_m_^sp^		[ΔH_m_/T_m_] × [ΔH_m_^sd^/ΔH_m_^sp^]^−1^		MACSi (at.%)	
OHS1	27.7	2090	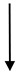	13.25	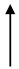	1.37	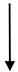	1653	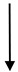	437	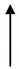	3.8	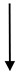	9.67	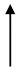	6.8	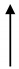
JZ3	29.1	2242		13		1.6		1823		419		4.35		8.11		4	
KZ4 (Cr)	28.2	2335		12.1		2.44		2060		275		7.5		4.96		1.9	

KZ4 = Nb-24Ti-18Si-5Cr [38], OHS1 = Nb-18Si-24Ti-5Al-5Cr-5Ge-5Sn [13] (nominal compositions).

**Table 7:** Phases in the diffusion zones of the alloys OHS1 [13], JZ2 [14], JZ3 and JZ3+ at 1200 °C. The symbol √ means that a phase is present in an alloy.

Phase	Alloy			
	OHS1	JZ2	JZ3	JZ3+
Nb_ss_	√	-	-	-
(Nb,W)_ss_	-	√ W/Ta = 0.7	√ W/Ta = 3.15	√ W/Ta = 3.64
A15	√	√ <Si> = 18.2	√ <Si> = 25.8	√ <Si> *
(Ti,Nb)_6_Sn_5_	√	-	-	-
Nb_5_Sn_2_Si	√ Nb/Ti = 1.95 <Si> = 37.6	-	-	-
NbGe_2_	-	√	-	-
Nb_5_(Si,Ge)_3_	√ <Si> = 37.9	√ RM/(Ti+Hf) = 38 <Si> = 37.7	√ RM/(Ti+Hf) = 30 <Si> = 37.8	√ RM/(Ti+Hf) = 33 <Si> = 38.5
W-rich Nb_5_(Si,Ge)_3_	-	√ RM/(Ti+Hf) = 51 <Si> = 35.2	-	-
Nb_5_Si_3_	√	√ RM/(Ti+Hf) = 6.2 <Si> = 37.9	√ RM/(Ti+Hf) = 4.8 <Si> = 37.3	√ RM/(Ti+Hf) = 2.9 <Si> = 39.2
Ti rich Nb_5_Si_3_	-	√ RM/(Ti+Hf) = 3. <Si> = 34.9	-	-
Nb_5_(Si,Sn)_3_	-	-	√ RM/(Ti+Hf) = 117 <Si> = 38.6	√ RM/(Ti+Hf) = 6.5 <Si> = 38.8
Sn rich layer	-	√	-	-
Ge rich layer	-	√	-	-

* see text, <Si> = Al+Ge+Si+Sn, RM = Nb, Ta, W.

**Table 8:** Comparison of calculated and experimental vol.% Nb_ss_, weight changes (ΔW/A, mg/cm^2^) at 800 and 1200 °C and macrosegregation of Si (MACSi, at.%).

Alloy	Nb_ss_^cal^	Nb_ss_^exp^	(ΔW/A)_cal_	(ΔW/A)_exp_	(ΔW/A)_cal_	(ΔW/A)_exp_	MACSi_cal_	MACSi_exp_
	As cast		800 °C		1200 °C		As cast	
JZ1	44.1	46.5	17	33.6	77	91	4.8	5.6
JZ2	34.8	34.5	12	28.9	54	72	5.2	4.9
JZ3	9.9	4.7	9.9	14.9	42	24.2	4.6	4
JZ3+	7.4	1.2	7.4	13.9	15	14	3.5	3.1

**Table 9:** The parameters VEC, Δχ and δ and the ratios sd/sp and Nb/(Ti+Hf) of the alloys JZ1, JZ2, JZ3 and JZ3+.

Alloy	VEC	Δχ	δ	Sd/Sp	Nb/(Ti+Hf)
JZ1	4.642	0.1745	8.93	2.92	4.41
JZ2	4.562	0.1859	9.14	2.39	3.95
JZ3	4.584	0.1942	9.1	2.24	3.11
JZ3+	4.546	0.1932	9.63	1.89	2.95

## Supplementary Materials

The following are available online at https://www.mdpi.com/1996-1944/13/17/3719/s1, Table S1: EDS analysis data (at.%) of the alloy JZ3-AC, Table S2: EDS analysis data (at.%) of the alloy JZ3-HT, Table S3: EDS analysis data (at.%) of the alloy [JZ3+]-AC, Table S4: EDS analysis data (at.%) of the alloy [JZ3+]-HT, Table S5: EDS analysis data (at.%) of phases in the alloy JZ3 after oxidation at 800 °C for 100 h, Table S6: EDS analysis data (at.%) of phases in the alloy JZ3 after oxidation at 1200 °C for 100 h, Table S7: EDS analysis data (at.%) of phases in the alloy JZ3+ after oxidation at 1200 °C for 100 h.

## Abbreviations

AC	as cast
BSE	back scattered electron image
CCA	complex concentrated alloy
DBTT	ductile to brittle transition temperature
DSC	differential scanning calorimetry
EDS	energy dispersive X-ray spectrometry
HEA	high entropy alloy
HT	heat treated
HV	Hardness Vickers
MACSi	macrosegregation of Si
MASC	metal and silicide composite
M_5_Si_3_	5-3 silicide of element M, where M is TM or RM
Nb_ss_	niobium solid solution
NICE	Niobium Intermetallic Composite Elaboration
RM	refractory metal
RCCA	refractory complex concentrated alloy
RHEA	refractory high entropy alloy
RMIC	refractory metal intermetallic composite
SEM	scanning electron microscope
TG	thermogravimetric analysis
TM	transition metal
VEC	valence electron concentration
XRD	X-ray diffraction
Δ	parameter related to atomic size
ΔW/A	weight change per unit area
Δχ	parameter related to electronegativity
Έ	creep rate (s^−1^)
ρ	density
<Si>	Al+Ge+Si+Sn
T_m_	melting point

## Appendix A

### Appendix A.1. Definition of the Parameters δ, Δχ and VEC

$\delta =100\sqrt{\overset{n}{\underset{n=1}{\sum }}c_{i}\;{\left(1-r_{i}/\bar{r}\right)}^{2}}$
$\Delta \chi =\sqrt{\overset{n}{\underset{n=1}{\sum }}c_{i}\;{\left(\chi_{i}-\bar{\chi }\right)}^{2}}$
$VEC=\overset{n}{\underset{n=1}{\sum }}c_{i}\;{\left(\mathrm{VEC}\right)}_{i}$
$\bar{r\;\;}=\overset{n}{\underset{n=1}{\sum }}c_{i}\;r_{i}$
$\bar{\chi }=\overset{n}{\underset{n=1}{\sum }}c_{i}\;\chi_{i}$

where c_i_, r_i_, χ_i_ and (VEC)_i_ respectively are atomic percentage, atomic radius, Pauling electronegativity and VEC of ith element.

### Appendix A.2. Alloys and Their Nominal Compositions (at.%)

JZ1 = Nb-12Ti-18Si-6Ta-2.5W-1Hf-2Sn-2Ge
JZ2 = Nb-12Ti-18Si-6Ta-2.5W-1Hf-5Sn-5Ge
KZ4 = Nb-24Ti-18Si-5Cr
KZ5 = Nb-24Ti-18Si-5Al-5Cr
KZ6 = Nb-24Ti-18Si-6Ta-5Al-5Cr
KZ7 = Nb-24Ti-18Si-5Al
KZ8 = Nb-24Ti-18Si-8Cr-4Al
MASC =Nb-25Ti-16Si-8Hf-2Al-2Cr
OHS1 = Nb-24Ti-18Si-5Al-5Cr-5Ge-5Sn
ZF5 = Nb-24Ti-18Si-5Al-5Ge
ZX8= Nb-24Ti-18Si-5Al-5Cr-5Sn

## References

[1] Balsone S.J., Bewlay B.P., Jackson M.R., Subramanian P.R., Zhao J.-C., Chatterjee A., Heffernan T.M., Hemker K.J., Dimiduk B.M., Clemens H., Darolia R., Inui M., Larsen J.M., Sikka V.K., Thomas M., Whittenberger J.D. (2001). Materials beyond superalloy-exploiting high temperature composites. Structural Intermetallics 2001.

[2] Senkov O.N., Miracle D.B., Chaput K.J. (2018). Development and exploration of refractory high entropy alloys—A review. J. Mater. Res..

[3] Bewlay B.P., Jackson M.R., Gigliotti M.F.X., Westbrook J.H., Fleischer R.L. (2002). Niobium Silicide High Temperature in Situ Composites. Intermetallic Compounds: Principles and Practice.

[4] Tsakiropoulos P. (2018). On Nb silicide based alloys: Alloy design and selection. Materials.

[5] Tsakiropoulos P. (2020). Alloys for application at ultra-high temperatures: Nb-silicide in situ composites-Challenges, breakthroughs and opportunities. Prog. Mater. Sci..

[6] Jackson M.R., Bewlay B.P., Briant C.L. (2002). Creep Resistant Nb-Silicide Based Two Phase Composites. U.S. Patent.

[7] Tsakiropoulos P. (2019). Alloys. U.S. Patent.

[8] Jackson M.R., Bewlay B.P., Zhao J.-C. (2002). Niobium Silicide Based Composites Resistant to Low Temperature Pesting. U.S. Patent.

[9] Menon E.S.K., Mendiratta M.G.D., Dimiduk M. (2002). Oxidation behavior of complex niobium based alloys. Niobium Science & Technology: Proceedings of the International Symposium Niobium 2001, held in Orlando, Florida, USA, 2–5 December 2001.

[10] Xu Z., Utton C., Tsakiropoulos P. (2020). A study of the effect of 5 at.% Sn on the microstructure and isothermal oxidation at 800 and 1200 °C of Nb-24Ti-18Si based alloys with Al and/or Cr additions. Materials.

[11] Li Z., Tsakiropoulos P. (2019). The effect of Ge addition on the oxidation of Nb-24Ti-18Si silicide based alloys. Materials.

[12] Knittel S., Mathieu S., Portebois L., Vilasi M. (2014). Effect of tin addition on Nb-Si based in situ composites. Part II: Oxidation behaviour. Intermetallics.

[13] Hernandez-Negrete O., Tsakiropoulos P. (2020). On the microstructure and isothermal oxidation at 800 and 1200 °C of the Nb-24Ti-18Si-5Al-5Cr-5Ge-5Sn (at.%) silicide based alloy. Materials.

[14] Zhao J., Utton C., Tsakiropoulos P. (2020). On the microstructure and properties of Nb-12Ti-18Si-6Ta-2.5W-1Hf (at.%) silicide based alloys with Ge and Sn additions. Materials.

[15] Schneibel J.H. (2005). Beyond Nickel-Base Superalloys. Processing and Fabrication of Advanced Materials XIII.

[16] Bewlay B.P., Jackson M.R., Zhao J.-C., Subramanian P.R., Mendiratta M.G., Lewandowski J.J. (2003). Ultrahigh temperature Nb-silicide based composites. MRS Bull..

[17] Simonenko E.P., Sevast’yanov D.V., Simonenko N.P. (2013). Promising ultra-high-temperature ceramic materials for aerospace applications. Russ. J. Inorg. Chem..

[18] Tang S., Hu C. (2017). Design, preparation and properties of carbon fiber reinforced ultra-high temperature ceramic composites for aerospace applications: A review. J. Mater. Sci. Technol..

[19] Tsakiropoulos P. (2018). On Nb silicide based alloys; Part II. J. Alloy. Compd..

[20] Bewlay B.P., Whiting P.W., Davis A.W., Briant C.L. (1999). Creep Mechanisms in Niobium-Silicide Based in-Situ Composites. Mat. Res. Soc. Symp. Proc..

[21] Prokoshkin D.A., Vasileva E.V., Samarin A.M. (1965). Alloys of Niobium.

[22] Kim J.-H., Tabaru T., Sakamoto M., Hanada S. (2004). Mechanical properties and fracture behaviour of an Nb_SS_/Nb_5_Si_3_ in-situ composite modified by Mo and Hf alloying. Mater. Sci. Eng..

[23] Guo H., Guo X. (2011). Microstructure evolution and room temperature fracture toughness of an integrally directionally solidified Nb-Ti-Si based ultrahigh temperature alloys. Scripta Mater..

[24] Xu Z., Utton C., Tsakiropoulos P. (2018). A study of the effect of 2 at.% Sn on the microstructure and isothermal oxidation at 80 and 1200 °C of Nb-24Ti-18Si based alloys with Al and/or Cr additions. Materials.

[25] Geng J., Tsakiropoulos P., Shao G. (2007). A thermo-gravimetric and microstructural study of the oxidation of Nb_ss_/Nb_5_Si_3_ based in situ composites with Sn addition. Intermetallics.

[26] Tsakiropoulos P. (2017). On the Nb silicide based alloys: Part I-The bcc Nb solid solution. J. Alloy. Compd..

[27] Grammenos I., Tsakiropoulos P. (2011). Study of the role of Hf, Mo and W additions in the microstructure of Nb-20Si silicide based alloy. Intermetallics.

[28] McCaughey C., Tsakiropoulos P. (2018). Type of primary Nb_5_Si_3_ and precipitation of Nb_ss_ in αNb_5_Si_3_ in a Nb-8.3Ti-21.1Si-5.4Mo-4W-0.7Hf (at.%) near eutectic Nb-silicide based alloy. Materials.

[29] MacKay R.A., Gabb T.P., Smialek J.L., Nathal M.V. (2009). Alloy Design Challenge: Development of Low Density Superalloys for Turbine Blade Applications.

[30] Begley R.T., Bechtold J.H. (1961). Effect of alloying on the mechanical properties of Niobium. J. Less Common Met..

[31] Menon E.S.K., Mendiratta M.G., Dimiduk D.M., Hemker K.J., Dimiduk D.M.H., Darolia C.R., Inui H.D., Larsen J.M., Sikka V.K., Thomas M., Whittenberger J.D. (2001). High temperature oxidation mechanisms in Nb-silicide bearing multicomponent alloys. Structural Intermetallics 2001.

[32] Tsakiropoulos P. (2018). On the alloying and properties of tetragonal Nb_5_Si_3_ in Nb-silicide based alloys. Materials.

[33] Fujikara M., Kasama A., Tanaka R., Hanada S. (2004). Effect of alloy chemistry on the high temperature strengths and room temperature fracture toughness of advanced Nb-based alloys. Mater. Trans..

[34] Tsakiropoulos P. (2014). On the macrosegregation of silicon in niobium silicide based alloys. Intermetallics.

[35] Li Z., Tsakiropoulos P. (2019). On the microstructure and hardness of the Nb-24Ti-18Si-5Al-5Cr-5Ge and Nb-24Ti-18Si-5Al-5Cr-5Ge-5Hf (at.%) silicide based alloys. Materials.

[36] Zelenitsas K., Tsakiropoulos P. (2006). Study of the role of Ta and Cr additions in the microstructure of Nb-Ti-Si-Al in situ composites. Intermetallics.

[37] Li Z., Tsakiropoulos P. (2013). The microstructures of Nb-18Si-5Al-5Ge and Nb-24Ti-18Si-5Al-5Ge in situ composites. J. Alloy. Compd..

[38] Zelenitsas K., Tsakiropoulos P. (2005). Study of the role of Cr and Al additions in the microstructure of Nb-Ti-Si in situ composites. Intermetallics.

[39] Okamoto H. (2000). Phase Diagrams for Binary Alloys: Desk Handbook.

[40] Grammenos I. (2008). Ultra-High Temperature Nb-Silicide Based Alloys. Ph.D. Thesis.

[41] Begley R.T., E Dalder N.C., Grobstein T., Chen C.S. (1994). Columbium alloy development at Westinghouse. Evolution of Refractory Metals and Alloys.

[42] Schlesinger M.E., Okamoto H., Gokhale A.B., Abbaschian R. (1993). The Nb-Si (Niobium-Silicon) System. J. Phase Equilibria.

[43] Toffolon C., Servant C., Gachon J.C., Sundman B. (2002). Reassessment of the Nb-Sn System. J. Phase Equilibria.

[44] Massalski T.B., Subramanian P.R., Okamoto H., Kacprzak L. (1990). Binary Alloy Phase Diagrams.

[45] Tsakiropoulos P. (2018). Alloying and properties of C14-NbCr_2_ and A15-Nb_3_X (X = Al,Ge,Si,Sn) in Nb-silicide based alloys. Materials.

[46] Sarath E., Menon K., Kim Y.-W., Corneiro T. (2004). Phase transformations and oxidation resistance of Nb-Ti-Si based alloys. Niobium for High Temperature Applications.

[47] Nelson J., Ghadyani M., Utton C., Tsakiropoulos P. (2018). A study of the effects of Al, Cr, Hf and Ti additions on the microstructure and oxidation of Nb-24Ti-18Si silicide based alloys. Materials.

[48] Westbrook J., Wood D. (1964). Pest degradation in beryllides, silicides, aluminides, and related compounds. J. Nucl. Mater..

[49] Zelenitsas K., Tsakiropoulos P. (2006). Effect of Al, Cr and Ta additions on the oxidation behaviour of Nb–Ti–Si in situ composites at 800 °C. Mater. Sci. Eng. A.

[50] Vellios N. (2007). Design of Nb-Silicide Based Alloys for Aero-Engines. Ph.D. Thesis.

[51] Bewlay B.P., Cretegny L., Jackson M.R., Subramanian P.R. (2006). Niobium Silicide Based Composites and Related Articles. U.S. Patent.

[52] Li Z., Tsakiropoulos P. (2012). Study of the effect of Cr and Ti additions in the microstructure of Nb-18Si-5Ge based in situ composites. Intermetallics.

[53] Bewlay B.P., Lipsitt H.A., Reeder W.J., Jackson M.R., Sutliff J.A., Ravi V.A., Srivatsan T.S., Moore J.J. (1994). Toughening mechanisms in directionally solidified Nb-Nb_3_Si-Nb_3_Si_5_ in-situ composites. Processing and Fabrication of Advanced Materials III.

[54] Sun Z., Guo X., Zhang C. (2012). Thermodynamic modeling of the Nb-rich corner in the Nb–Si–Sn system. CALPHAD Comput. Coupling Phase Diagr. Thermochem..

[55] Papadimitriou I., Utton C., Tsakiropoulos P. (2019). Ab initio study of ternary W_5_Si_3_ type TM_5_Sn_2_X compounds (TM = Nb, Ti, X = Al, Si). Materials.

[56] Yurchenko N., Panina E., Zherebtsov S., Salischev G., Stepanov N. (2018). Oxidation behaviour of refractory AlNbTiVZr_0.25_ high entropy alloys. Materials.

