# Supplementary material for: On the Microstructure and Properties of Nb-12Ti-18Si-6Ta-5Al-5Cr-2.5W-1Hf (at.%) Silicide-Based Alloys with Ge and Sn Additions
Source: Materials (Basel). 2020 Aug 22;13(17):3719. doi: 10.3390/ma13173719 (PMC7504593; doi:10.3390/ma13173719)
Supplement: Supplementary file 1 [file materials-13-03719-s001.pdf]

Supplementary Materials: On the Microstructure and Properties of Nb-12Ti-18Si-6Ta-5Al-5Cr-2.5W-1Hf (at.%) Silicide-Based Alloys with Ge and Sn Additions

Jiang Zhao, Claire Utton and Panos Tsakiropoulos

Table S1. EDS analysis data (at.%) of the alloy JZ3-AC.

	Nb	Ti	Si	Ta	W	Sn	Ge	Hf	Al	Cr
Top[a]	41.7 ± 0.6	12.9 ± 0.7	17.1 ± 1.5	6.2 ± 0.5	2.9 ± 0.4	3.4 ± 0.3	4.7 ± 0.6	1.0 ± 0.1	4.6 ± 0.4	5.5 ± 1.0
	41.0–42.5	12.2–13.9	15.3–18.8	6.0–6.5	2.5–3.3	2.9–3.5	4.0–5.6	0.9–1.2	4.1–5.1	4.6–6.7
Bulk[a]	41.5 ± 0.7	12.2 ± 0.4	18.5 ± 1.3	5.9 ± 0.5	2.7 ± 0.5	3.5 ± 0.3	4.9 ± 0.4	1.1 ± 0.1	4.7 ± 0.4	5.0 ± 0.5
	40.6–42.4	11.8–12.9	16.4–19.3	5.2–6.5	2.3–3.6	3.1–4.0	4.6–5.5	0.9–1.2	4.3–5.2	4.3–5.5
Bottom[a]	41.8 ± 0.3	12.5 ± 0.3	16.9 ± 1.1	5.9 ± 0.3	2.7 ± 0.2	4.2 ± 0.4	4.7 ± 0.3	1.0 ± 0.2	4.9 ± 0.3	5.4 ± 0.4
	41.0–41.9	12.0–12.7	15.9–18.3	5.6–6.3	2.4–2.9	3.7–4.8	4.1–5.1	0.7–1.2	4.6–5.4	5.0–5.9
Nbss[b]	26.4 ± 2.4	31.4 ± 1.9	2.4 ± 1.2	3.6 ± 0.3	1.5 ± 0.1	3.5 ± 0.8	1.2 ± 0.3	2.3 ± 0.5	8.6 ± 1.6	19.1 ± 5.3
	22.9–28.8	29.0–33.2	1.6–4.4	3.2–4.0	1.3–1.6	2.2–4.4	0.9–1.5	1.8–3.0	6.3–10.0	14.1–26.9
Nbss[c]	40.6 ± 0.7	14.1 ± 0.7	2.9 ± 1.1	10.4 ± 0.5	10.9 ± 1.0	2.5 ± 0.1	0.5	0.3	5.6 ± 0.5	12.2 ± 0.4
	39.5–41.1	13.4–15.0	1.0–3.6	9.9–11.2	9.4–12.3	2.4–2.5			5.1–6.3	11.7–12.8
Nb5Si3	42.6 ± 0.4	9.5 ± 0.3	27.8 ± 1.5	6.0 ± 0.2	1.2 ± 0.1	1.5 ± 0.3	6.6 ± 0.3	0.6	2.8 ± 0.6	1.4 ± 0.1
	42.3–43.1	9.2–9.9	26.2–29.8	5.8–6.2	1.0–1.4	1.2–1.9	6.2–7.0		2.3–3.5	1.1–1.5
Ti-rich Nb5Si3	37.3 ± 2.3	15.9 ± 1.9	23.1 ± 1.6	4.5 ± 0.4	0.6	2.0 ± 0.1	6.9 ± 0.1	1.6 ± 0.5	4.6 ± 0.2	3.5 ± 1.4
	34.4–39.7	14.1–18.8	20.5–24.8	4.0–5.0		1.9–2.1	6.7–7.1	1.1–2.3	4.3–4.8	2.5–5.8
A15	50.9 ± 0.2	9.5 ± 0.3	5.2 ± 0.7	9.2 ± 0.2	7.1 ± 0.2	7.3 ± 0.2	2.2 ± 0.6	0.5	4.7 ± 0.4	3.4 ± 0.2
	50.6–51.1	9.1–9.9	4.5–6.2	9.0–9.5	6.8–7.2	7.1–7.7	1.7–3.1		4.2–5.2	3.1–3.7
Ti-rich A15[d]	45.3 ± 0.4	15.4 ± 0.2	4.2 ± 0.3	7.2 ± 0.3	4.9 ± 0.2	7.0 ± 0.2	1.7 ± 0.2	0.6	6.5 ± 0.2	7.2 ± 0.3
	45.0–46.0	15.0–15.6	3.8–4.5	6.8–7.4	4.7–5.1	6.8–7.2	1.6–2.0		6.2–6.8	6.7–7.5
Cr-rich A15[d]	26.8 ± 1.1	25.6 ± 1.2	9.8 ± 2.4	3.2 ± 0.5	1.0 ± 0.4	3.0 ± 0.4	4.3 ± 1.4	2.6 ± 0.3	7.2 ± 0.7	16.5 ± 3.2
	25.2–29.2	23.5–27.4	6.5–13.3	2.8–3.6	0.6–1.2	2.5–3.3	2.9–6.7	2.1–3.1	6.2–8.3	12.2–21.9
C14-Cr2Nb	20.1 ± 0.3	9.0 ± 0.7	8.8 ± 1.1	6.4 ± 0.4	3.2 ± 0.2	0.3	1.1 ± 0.1	1.7 ± 0.1	4.6 ± 0.2	44.8 ± 0.6
	19.8–20.5	8.2–10.0	7.0–9.9	5.8–6.9	2.9–3.4		1.0–1.2	1.6–1.9	4.3–4.8	44.0–45.6

[a] Large area analysis. [b] Solid solution rich in Ti and Cr. This solid solution was present with a very small vol%.in the top and bulk of the as cast button. [c] Solid solution present only in the bottom of the as cast button. [d] A15 phase rich in Ti or Cr was present in the bulk of the as cast button.

Table S2. EDS analysis data (at.%) of the alloy JZ3-HT.

	Nb	Ti	Si	Ta	W	Sn	Ge	Hf	Al	Cr
Large area	41.7 ± 0.4	12.8 ± 0.1	18.3 ± 1.0	5.7 ± 0.4	2.5 ± 0.2	3.4 ± 0.5	5.2 ± 0.2	0.8 ± 0.2	4.8 ± 0.1	4.8 ± 0.3
	41.2–42.2	12.7–12.8	16.8–19.4	5.4–6.3	2.2–2.7	2.8–2.9	5.0–5.4	0.5–1.1	4.5–4.9	4.3–5.1
Nbss	43.1 ± 0.5	9.2 ± 0.3	2.1 ± 1.0	12.4 ± 0.4	17.0 ± 0.2	1.4 ± 0.1	0.3	–	3.5 ± 0.1	11.0 ± 0.2
	42.5–43.4	8.8–9.6	1.2–3.3	11.9–12.8	16.0–17.3	1.3–1.6			3.4–3.6	10.7–11.1
Nb5Si3	41.8 ± 0.5	9.4 ± 0.6	28.5 ± 1.0	5.7 ± 0.3	1.2 ± 0.2	1.2 ± 0.1	6.2 ± 0.2	0.7	2.7 ± 0.4	2.6 ± 0.4
	41.1–42.5	8.9–10.2	27.6–29.8	5.4–6.0	0.9–1.4	1.1–1.4	6.0–6.4		2.3–3.4	2.1–3.1
Ti-rich Nb5Si3	37.4 ± 3.3	16.2 ± 3.4	25.2 ± 0.9	4.2 ± 0.5	0.3	1.4 ± 0.7	7.1 ± 0.4	0.7 ± 0.5	4.7 ± 0.5	2.8 ± 0.1
	33.6–40.2	13.4–20.0	24.3–26.4	3.4–4.8		0.6–2.1	6.5–7.6	0.3–1.6	4.3–5.3	2.6–2.9
A15	49.1 ± 0.4	11.9 ± 0.2	5.2 ± 0.6	7.1 ± 0.3	5.3 ± 0.1	8.0 ± 0.2	1.6 ± 0.2	–	5.9 ± 0.1	5.9 ± 0.2
	48.4–49.5	11.7–12.2	4.6–6.0	6.7–7.4	5.2–5.4	7.7–8.2	1.4–1.9		5.7–6.0	5.6–6.2
C14-Cr2Nb	20.3 ± 0.3	4.2 ± 0.1	11.2 ± 0.5	8.2 ± 0.2	4.0 ± 0.1	0.2	0.8 ± 0.1	–	3.0 ± 0.2	48.1 ± 0.3
	19.8–20.7	4.1–4.4	10.6–11.2	7.9–8.5	3.8–4.1		0.6–0.9		2.8–3.3	47.7–48.4

Table 3. EDS analysis data (at.%) of the alloy [JZ3+]-AC.

	Nb	Ti	Si	Ta	W	Sn	Ge	Hf	Al	Cr
Top [a]	39.2 ± 0.7	12.5 ± 0.2	20.0 ± 1.0	5.6 ± 0.4	2.1 ± 0.3	5.0 ± 0.3	5.1 ± 0.1	0.8 ± 0.1	4.6 ± 0.3	5.1 ± 0.4
	38.0–39.8	12.1–12.7	18.3–20.6	5.0–6.0	1.9–2.6	4.6–5.2	4.9–5.3	0.6–1.0	4.3–5.1	4.5–5.6
Bulk [a]	39.8 ± 0.3	12.3 ± 0.4	19.1 ± 0.9	5.8 ± 0.3	2.6 ± 0.3	5.3 ± 0.4	4.6 ± 0.2	0.8 ± 0.1	4.5 ± 0.2	5.2 ± 0.2
	39.3–40.1	11.9–12.8	17.7–19.9	5.6–6.0	2.2–3.1	4.6–5.8	4.5–5.0	0.7–0.9	4.3–4.7	4.9–5.2
Bottom [a]	37.7 ± 0.4	12.6 ± 0.2	20.1 ± 0.7	5.6 ± 0.1	2.2 ± 0.3	6.0 ± 0.3	4.8 ± 0.1	0.9 ± 0.1	4.7 ± 0.2	5.4 ± 0.7
	37.4–38.0	12.2–12.8	19.0–20.8	5.4–5.7	2.0–2.8	5.7–6.3	4.7–5.0	0.8–1.0	4.5–5.0	4.9–6.4
(Nb,W)ss [b]	31.2 ± 1.5	4.9 ± 0.3	1.7 ± 1.3	15.2 ± 0.2	35.3 ± 1.4	1.8 ± 0.2	0.3	0.1	2.4 ± 0.2	7.1 ± 0.5
	29.4–32.8	4.5–5.3	0.6–3.6	14.8–15.5	33.8–37.5	1.5–2.1			2.1–2.7	6.3–7.5
Nb5Si3	42.0 ± 0.3	9.2 ± 0.2	30.6 ± 0.6	6.3 ± 0.2	1.3 ± 0.1	1.3 ± 0.0	5.5 ± 0.2	0.5	1.9 ± 0.1	1.4 ± 0.2
	41.7–42.3	8.9–9.4	29.8–31.3	6.1–6.6	1.3–1.4	1.2–1.5	5.2–5.6		1.8–2.0	1.2–1.6
Ti-rich Nb5Si3	32.7 ± 2.0	21.4 ± 3.0	16.7 ± 2.0	2.9 ± 0.4	0.4	8.1 ± 0.9	5.3 ± 0.5	1.6 ± 0.2	7.3 ± 1.1	3.6 ± 0.3
	29.4–34.1	18.6–26.2	14.3–19.4	2.3–3.3		7.3–9.5	4.6–5.7	1.4–1.8	6.5–9.1	3.0–3.9
A15 phase	46.3 ± 0.4	11.3 ± 0.2	5.2 ± 0.5	7.8 ± 0.3	7.3 ± 0.2	10.4 ± 0.2	1.3 ± 0.1	0.3	4.8 ± 0.0	5.3 ± 0.4
	45.4–46.6	11.1–11.6	4.5–5.6	7.4–8.1	7.0–7.6	10.0–10.7	1.2–1.5		4.8–4.9	5.0–5.9
Ti-rich A15	39.9 ± 01.9	19.3 ± 3.5	2.8 ± 0.5	4.9 ± 0.3	4.0 ± 1.1	12.6 ± 0.9	0.9 ± 0.2	0.5	6.4 ± 0.6	8.7 ± 1.0
	37.3–42.2	13.9–23.3	2.1–3.2	4.1–7.0	3.1–5.9	11.2–13.5	0.6–1.2		5.7–6.9	7.8–9.8
C14-Cr2Nb	20.5 ± 0.6	6.6 ± 0.6	8.7 ± 0.5	6.9 ± 0.5	4.4 ± 0.5	0.6	1.0 ± 0.1	1.1 ± 0.1	6.0 ± 0.6	44.2 ± 0.7
	20.0–21.4	5.8–7.2	8.2–9.5	6.3–7.7	3.9–5.1		0.6–1.1	0.8–1.2	5.2–6.7	43.5–45.1

[a] Large area analysis. [b] Solid solution formed only in the bottom of the as cast button.

Table S4. EDS analysis data (at.%) of the alloy [JZ3+]-HT.

	Nb	Ti	Si	Ta	W	Sn	Ge	Hf	Al	Cr
Large area	39.3 ± 0.3	12.3 ± 0.2	20.7 ± 1.1	5.7 ± 0.2	2.0 ± 0.3	4.8 ± 0.3	5.1 ± 0.1	0.8 ± 0.1	4.6 ± 0.3	4.7 ± 0.2
	39.1–39.9	12.2–12.5	19.4–21.9	5.5–6.0	1.7–2.3	4.5–5.1	5.0–5.2	0.6–0.8	4.2–4.9	4.4–4.9
(Nb,W)ss	30 ± 0.7	3.9 ± 0.4	2.1 ± 0.1	15.1 ± 0.6	39.8 ± 0.6	0.4	–	–	1.5 ± 0.2	7.1 ± 0.4
	29.0–30.8	3.4–4.3	0.2–3.1	14.3–16.0	38.8–40.2				1.4–1.8	6.7–7.4
Nb5Si3	39.2 ± 0.4	13.0 ± 0.2	23.8 ± 0.9	4.9 ± 0.3	0.8 ± 0.1	4.2 ± 0.2	5.6 ± 0.2	0.9 ± 0.1	4.6 ± 0.3	3.0 ± 0.2
	38.5–39.6	12.7–13.3	22.7–25.0	4.6–5.3	0.7–1.0	3.9–4.4	5.4–5.9	0.7–1.0	4.2–5.0	2.9–3.4
Ti-rich Nb5Si3	34.2 ± 0.2	16.0 ± 0.5	25.5 ± 0.9	4.9 ± 0.5	0.5	1.3 ± 0.5	6.5 ± 0.3	3.2 ± 0.4	5.1 ± 0.2	2.8 ± 0.2
	33.8–34.5	15.4–16.6	24.0–26.4	4.4–5.6		1.0–2.2	6.2–6.9	2.5–3.6	4.8–5.3	2.6–3.1
A15	48.1 ± 0.4	11.6 ± 0.2	2.8 ± 0.6	8.2 ± 0.2	6.5 ± 0.2	12.2 ± 0.3	0.8 ± 0.2	-	4.7 ± 0.1	5.1 ± 0.3
	47.6–48.9	11.4–11.8	1.8–3.5	8.0–8.5	6.3–6.9	11.9–12.6	0.6–1.0		4.7–4.9	4.9–5.5
C14-Cr2Nb	19.2 ± 0.6	3.4 ± 0.3	11.4 ± 0.3	9.6 ± 0.2	5.2 ± 0.3	0.4	0.7	0.5	3.1 ± 0.2	46.5 ± 1.2
	18.7–20.3	3.1–3.7	11.2–11.9	9.4–9.8	4.7–5.3				2.8–3.4	44.7–48.1

Table S5. EDS analysis data (at.%) of phases in the alloy JZ3 after oxidation at 800 °C for 100 h.

Area and Phase	O	Nb	Ti	Si	Ta	W	Sn	Ge	Hf	Al	Cr
					Scale						
Nb-rich oxide	73.6 ± 0.6<br>72.1–74.4	14.5 ± 0.2<br>14.3–14.7	2.9 ± 0.3<br>2.5–3.2	1.5 ± 0.4<br>1.1–2.1	2.5 ± 0.1<br>2.3–2.6	1.7 ± 0.1<br>1.6–1.8	0.4	0.5	–	1.3 ± 0.1<br>1.1–1.4	1.2 ± 0.2<br>0.9–1.5
Si-rich oxide	72.3 ± 1.2<br>70.3–73.4	12.5 ± 0.6<br>12.0–13.3	2.9 ± 0.4<br>2.5–3.4	8.1 ± 0.3<br>7.7–8.5	1.6 ± 0.1<br>1.5–1.7	0.3	–	0.6	0.3	1.0 ± 0.1<br>0.9–1.2	0.3
					Bulk						
Nb5Si3	–	43.5 ± 0.4<br>42.9–44.0	9.6 ± 0.4<br>9.2–10.3	26.8 ± 0.8<br>25.7–27.9	6.0 ± 0.3<br>5.7–6.2	1.0 ± 0.2<br>0.8–1.3	1.7 ± 0.5<br>1.2–2.3	6.1 ± 0.3<br>5.7–6.5	0.7	3.2 ± 0.3<br>2.9–3.6	1.4 ± 0.1<br>1.4–1.5
Ti-rich Nb5Si3	–	40.9 ± 0.2<br>40.7–41.1	13.1 ± 0.6<br>12.3–13.8	24.2 ± 0.5<br>23.4–24.8	5.1 ± 0.2<br>4.8–5.4	0.7	2.2 ± 0.1<br>2.0–2.2	6.1 ± 0.2<br>5.9–6.4	1.3 ± 0.2<br>1.0–1.4	4.2 ± 0.1<br>4.1–4.3	2.3 ± 0.1<br>2.2–2.4
A15	–	52.8 ± 0.5<br>52.1–53.4	9.0 ± 0.8<br>8.4–10.0	5.2 ± 0.7<br>4.2–6.0	9.5 ± 0.7<br>9.0–10.6	6.4 ± 0.7<br>5.8–7.4	7.3 ± 0.1<br>7.1–7.4	1.9 ± 0.3<br>1.7–2.2	0.3	4.4 ± 0.3<br>4.1–4.9	3.1 ± 0.4<br>2.7–3.6
Ti-rich A15	–	49.6 ± 0.7<br>48.8–50.6	12.0 ± 0.9<br>11.1–13.5	5.5 ± 0.7<br>4.5–6.3	8.0 ± 0.3<br>7.6–8.4	4.9 ± 0.5<br>4.4–5.7	7.2 ± 0.1<br>7.0–7.3	2.1 ± 0.3<br>1.7–2.4	0.4	5.3 ± 0.2<br>5.0–5.7	5.0 ± 0.8<br>3.9–5.9
Nbss	–	47.9 ± 1.1<br>46.8–49.2	8.9 ± 1.0<br>7.7–10.3	3.4 ± 0.6<br>2.3–3.6	13.0 ± 0.6<br>12.4–13.8	13.3 ± 0.8<br>12.2–14.2	2.2 ± 0.2<br>2.0–2.5	1.3 ± 0.3<br>0.9–1.8	–	3.9 ± 0.3<br>3.5–4.2	6.0 ± 1.1<br>4.6–7.5
C14-Cr2Nb	–	21.8 ± 1.1<br>20.0–22.9	9.6 ± 1.2<br>8.5–10.8	9.1 ± 0.4<br>8.6–9.7	6.3 ± 0.6<br>5.6–7.1	2.3 ± 0.3<br>2.0–2.7	0.4	1.4 ± 0.3<br>1.1–1.8	1.7 ± 0.1<br>1.5–1.9	4.7 ± 0.3<br>4.2–5.0	42.8 ± 2.0<br>41.4–46.2

Table S6. EDS analysis data (at.%) of phases in the alloy JZ3 after oxidation at 1200 ^{o}C for 100 h.

	Phase	O	Nb	Ti	Si	Ta	W	Sn	Ge	Hf	Al	Cr
Oxide Scale	Nb-rich Oxide	72.5 ± 0.7<br>71.6–73.2	18.9 ± 1.2<br>17.3–20.4	2.6 ± 0.6<br>1.9–3.3	1.2 ± 0.3<br>0.8–1.7	2.9 ± 0.8<br>2.1–4.2	0.7	–	–	0.2	0.6	0.4
	Ti-rich Oxide	71.4 ± 0.3<br>71.1–71.8	10.7 ± 0.9<br>9.4–11.8	8.5 ± 0.8<br>7.4–9.6	0.5	1.5 ± 0.2<br>1.3–1.7	0.6	–	–	0.3	2.5 ± 0.1<br>2.5–2.7	4.2 ± 0.2<br>3.8–4.4
Diffusion Zone	Nb5Si3	–	43.7 ± 0.7<br>43.2–44.1	9.7 ± 0.1<br>9.6–9.8	25.8 ± 1.3<br>24.5–27.6	6.0 ± 0.4<br>5.4–6.4	1.1 ± 0.2<br>0.8–1.2	1.9 ± 0.1<br>1.8–2.1	6.0 ± 0.3<br>5.6–6.4	0.8 ± 0.1<br>0.6–0.9	3.6 ± 0.2<br>3.4–3.8	1.4 ± 0.3<br>1.1–1.8
	Nb5(Si,Sn,)3	–	51.5 ± 2.0<br>47.7–52.6	0.5	15.0 ± 1.9<br>11.6–16.3	6.4 ± 0.7<br>5.5–7.1	0.4	15.4 ± 4.3<br>12.7–22.8	8.2 ± 1.4<br>6.3–9.9	–	–	2.6 ± 0.3<br>2.1–2.9
	Nb5(Si1-x,Gex)3	–	50.9 ± 1.0<br>49.4–51.9	2.1 ± 0.9<br>1.0–3.1	10.9 ± 6.1<br>5.8–17.9	6.1 ± 0.2<br>5.9–6.3	1.8 ± 0.8<br>0.7–2.8	0.4	26.1 ± 4.8<br>20.8–30.6	–	0.4	1.2 ± 0.9<br>0.7–2.8
	A15	–	58.1 ± 0.4<br>57.3–58.2	0.9 ± 0.3<br>0.5–1.2	3.2 ± 0.2<br>3.0–3.5	8.4 ± 0.5<br>7.7–9.1	4.0 ± 0.5<br>3.2–4.5	21.3 ± 0.3<br>20.8–21.6	0.7	–	06	2.9 ± 0.3<br>2.6–3.2
	(Nb,,W)ss	–	15.5 ± 2.7<br>13.8–20.1	0.5	1.6 ± 1.0<br>0–2.5	18.3 ± 1.3<br>16.8–20.2	57.7 ± 2.1<br>54.5–60.2	0.9 ± 0.4<br>0.5–1.5	3.0 ± 0.8<br>2.1–4.2	–	–	2.5 ± 0.7<br>1.5–3.2
Bulk	Nb5Si3	–	43.5 ± 0.5<br>43.0–44.4	10.0 ± 0.7<br>9.2–10.8	26.8 ± 0.9<br>26.0–27.9	5.3 ± 0.6<br>4.6–6.1	0.9 ± 0.2<br>0.6–1.1	2.0 ± 0.4<br>1.5–2.5	5.6 ± 0.3<br>5.4–6.0	0.8 ± 0.3<br>0.4–1.1	3.6 ± 0.4<br>3.1–4.2	1.5 ± 0.1<br>1.3–1.6
	Ti-rich Nb5Si3	–	39.0 ± 0.4<br>38.5–39.5	13.9 ± 0.6<br>13.2–14.6	23.6 ± 0.7<br>22.9–24.7	4.9 ± 0.3<br>4.4–5.2	0.9 ± 0.1<br>0.8–1.0	3.0 ± 0.1<br>2.9–3.1	6.5 ± 0.2<br>6.1–6.7	1.1 ± 0.2<br>0.8–1.4	4.8 ± 0.2<br>4.5–4.9	2.4 ± 0.1<br>2.2–2.5
	A15	–	48.2 ± 0.9<br>47.0–49.0	12.4 ± 0.9<br>11.4–13.7	3.5 ± 1.0<br>2.8–5.3	8.4 ± 0.6<br>7.8–9.5	6.5 ± 0.5<br>5.8–7.1	8.5 ± 0.2<br>8.2–8.7	1.8 ± 0.2<br>1.5–2.1	0.3	5.9 ± 0.6<br>5.4–6.4	4.5 ± 1.1<br>3.2–5.7
	C14-Cr2Nb	–	19.9 ± 1.3<br>18.2–21.0	4.8 ± 0.4<br>4.4–5.3	10.4 ± 0.8<br>9.2–11.5	8.9 ± 0.5<br>8.4–9.5	3.4 ± 0.5<br>2.8–4.0	-	0.8 ± 0.3<br>0.5–1.3	1.4 ± 0.2<br>1.2–1.6	2.9 ± 0.2<br>2.6–3.1	47.6 ± 1.3<br>45.9–49.1

Table S7. EDS analysis data (at.%) of phases in the alloy JZ3+ after oxidation at 1200 °C for 100 h.

	Phase	O	Nb	Ti	Si	Ta	W	Sn	Ge	Hf	Al	Cr
Oxide Scale	Nb-rich Oxide	73.4 ± 1.1<br>71.6–74.8	17.2 ± 1.2<br>15.6–18.7	3.6 ± 0.6<br>2.6–3.8	1.3 ± 0.4<br>0.6–1.6	2.8 ± 0.4<br>2.2–3.1	0.9 ± 0.4<br>0.3–1.3	–	–	-	0.5	0.3
	Ti-rich Oxide	72.5 ± 0.3<br>72.1–72.9	8.6 ± 1.1<br>7.3–9.9	9.8 ± 1.7<br>7.6–11.8	0.5	1.5 ± 0.2<br>1.3–1.7	0.5	–	–	0.3	2.9 ± 0.6<br>2.4–3.1	3.4 ± 0.8<br>2.6–4.5
Diffusion Zone	Nb5Si3	–	42.2 ± 0.4<br>41.7–42.6	8.9 ± 0.4<br>8.4–9.4	30.6 ± 1.1<br>29.4–31.1	6.8 ± 0.3<br>6.4–7.1	1.4 ± 0.2<br>1.2–1.6	1.8 ± 0.7<br>1.0–2.7	5.4 ± 0.2<br>5.2–5.8	0.8 ± 0.2<br>0.6–1.1	1.4 ± 0.4<br>0.8–2.0	0.8 ± 0.2<br>0.5–1.0
	Nb5(Si,Sn)3	–	45.2 ± 0.7<br>44.4–45.8	7.5 ± 1.2<br>6.1–9.2	16.7 ± 1.6<br>14.4–18.9	5.0 ± 0.4<br>4.5–5.5	0.8 ± 0.7<br>0.3–2.0	15.4 ± 2.5<br>12.3–19.1	4.6 ± 0.4<br>3.9–5.1	0.4	2.1 ± 1.0<br>0.9–3.4	2.9 ± 0.4<br>2.5–3.5
	Nb5(Si1-x,Gex)3	–	49.2 ± 1.7<br>47.9–51.4	1.5 ± 0.9<br>0.9–3.0	17.9 ± 2.4<br>14.6–21.3	7.7 ± 0.7<br>7.1–8.8	2.5 ± 0.6<br>1.5–3.1	0.7	18.7 ± 2.4<br>15.3–21.2	0.3	1.2 ± 1.3<br>0.2–2.7	0.3
	(Nb,Ta,W)ss	–	14.7 ± 2.9<br>12.2–19.1	0.3	0.4	16.7 ± 2.0<br>13.5–19.1	60.7 ± 0.4<br>60.4–61.2	0.6	3.2 ± 0.6<br>2.2–3.8	–	–	3.4 ± 0.7<br>2.3–4.1
Bulk	Nb5Si3	–	41.6 ± 0.7<br>40.7–42.6	9.2 ± 0.5<br>8.4–9.8	29.6 ± 1.4<br>28.1–31.3	6.9 ± 0.5<br>6.3–7.6	1.3 ± 0.1<br>1.2–1.5	1.6 ± 0.3<br>1.3–1.9	5.5 ± 0.5<br>4.6–6.0	0.8 ± 0.2<br>0.5–1.0	2.2 ± 0.4<br>1.5–2.6	1.4 ± 0.2<br>1.1–1.6
	Ti-rich Nb5Si3	–	35.8 ± 0.5<br>35.0–36.2	19.5 ± 1.1<br>18.7–21.0	14.9 ± 1.9<br>13.1–17.9	3.7 ± 0.5<br>3.2–4.2	0.8 ± 1.2<br>0.5–1.2	9.3 ± 1.0<br>8.2–10.6	4.8 ± 0.3<br>4.5–5.3	1.7 ± 0.2<br>1.5–2.0	7.4 ± 0.5<br>7.2–8.1	2.0 ± 0.5<br>1.4–2.6
	A15	–	45.8 ± 0.5<br>45.3–46.5	12.4 ± 1.0<br>11.3–13.6	2.1 ± 0.3<br>1.6–2.4	7.9 ± 0.5<br>7.2–8.4	8.2 ± 0.4<br>7.9–8.9	10.4 ± 0.4<br>10.0–11.1	1.5 ± 0.5<br>1.0–2.0	0.4	6.3 ± 0.4<br>5.6–6.7	5.0 ± 0.7<br>4.3–5.9
	C14-Cr2Nb	–	20.1 ± 0.7<br>19.4–21.0	3.9 ± 0.6<br>3.3–4.6	10.4 ± 1.0<br>8.6–11.2	9.6 ± 0.4<br>9.2–10.2	4.5 ± 0.4<br>4.0–5.1	0.3	1.3 ± 0.1<br>1.2–1.4	1.2 ± 0.2<br>1.0–1.4	5.4 ± 0.4<br>4.9–6.0	43.4 ± 0.6<br>42.4–44.0
